# How Chemical Nature of Fixed Groups of Anion-Exchange Membranes Affects the Performance of Electrodialysis of Phosphate-Containing Solutions?

**DOI:** 10.3390/polym15102288

**Published:** 2023-05-12

**Authors:** Natalia Pismenskaya, Olesya Rybalkina, Ksenia Solonchenko, Evgeniia Pasechnaya, Veronika Sarapulova, Yaoming Wang, Chenxiao Jiang, Tongwen Xu, Victor Nikonenko

**Affiliations:** 1 Russian Federation, Kuban State University, 149, Stavropolskaya Str., 350040 Krasnodar, Russia; n_pismen@mail.ru (N.P.); olesia93rus@mail.ru (O.R.); sol.ksenia17@yandex.ru (K.S.); evgpasechnaya@yandex.ru (E.P.); vsarapulova@gmail.com (V.S.); 2 Anhui Provincial Engineering Laboratory of Functional Membrane Science and Technology, Department of Applied Chemistry, School of Chemistry and Materials Science, University of Science and Technology of China, Hefei 230026, China; ymwong@ustc.edu.cn (Y.W.); jcx11@ustc.edu.cn (C.J.); twxu@ustc.edu.cn (T.X.)

**Keywords:** anion exchange membrane, structure, weakly basic fixed groups, phosphates, bound species, conductivity, current–voltage curve, proton generation, electrodialysis, energy consumption

## Abstract

Innovative ion exchange membranes have become commercially available in recent years. However, information about their structural and transport characteristics is often extremely insufficient. To address this issue, homogeneous anion exchange membranes with the trade names ASE, CJMA-3 and CJMA-6 have been investigated in Na_x_H_(3−x)_PO_4_ solutions with pH 4.4 ± 0.1, 6.6 and 10.0 ± 0.2, as well as NaCl solutions with pH 5.5 ± 0.1. Using IR spectroscopy and processing the concentration dependences of the electrical conductivity of these membranes in NaCl solutions, it was shown that ASE has a highly cross-linked aromatic matrix and mainly contains quaternary ammonium groups. Other membranes have a less cross-linked aliphatic matrix based on polyvinylidene fluoride (CJMA-3) or polyolefin (CJMA-6) and contain quaternary amines (CJMA-3) or a mixture of strongly basic (quaternary) and weakly basic (secondary) amines (CJMA-6). As expected, in dilute solutions of NaCl, the conductivity of membranes increases with an increase in their ion-exchange capacity: CJMA-6 < CJMA-3 << ASE. Weakly basic amines appear to form bound species with proton-containing phosphoric acid anions. This phenomenon causes a decrease in the electrical conductivity of CJMA-6 membranes compared to other studied membranes in phosphate-containing solutions. In addition, the formation of the neutral and negatively charged bound species suppresses the generation of protons by the “acid dissociation” mechanism. Moreover, when the membrane is operated in overlimiting current modes and/or in alkaline solutions, a bipolar junction is formed at the CJMA- 6/depleted solution interface. The CJMA-6 current-voltage curve becomes similar to the well-known curves for bipolar membranes, and water splitting intensifies in underlimiting and overlimiting modes. As a result, energy consumption for electrodialysis recovery of phosphates from aqueous solutions almost doubles when using the CJMA-6 membrane compared to the CJMA-3 membrane.

## 1. Introduction

Ion-exchange membranes, as a rule, consist of high molecular weight polymers whose side chains of which contain polar fixed groups. Acid residues of polybasic acids (sulfonic, phosphoric, carboxylic, etc.), as well as primary, secondary, tertiary, quaternary amines, imidazole cations, guanidinium cations, are most often these groups [1,2]. Hydration and dissociation of these groups cause the formation of a system of through pores if the ion-exchange polymer is in an aqueous solution. Pore walls become electrically charged [3,4,5]. Ions that have the same charge as the dissociated fixed groups are called coions. They are excluded from the membrane due to the Donnan effect [6]. That is why the ion-exchange membrane is able to selectively transfer counterions: cations (pore walls have a negative charge) or anions (pore walls have a positive charge) under the action of a concentration gradient and/or a potential drop. Until recently, relatively few polymers have been used to form ion exchange membranes. First, it is polystyrene cross-linked with divinylbenzene, which is obtained by monomers polymerization [7]. Subsequent sulfonation or amination imparts ion-exchange properties to this material. For example, these are Neosepta AMX (Astom, Japan), Ralex AMH MH (MEGA, Czech Republic), MA-41 (Shchekinoazot, Russia), etc. In addition, these are polymers that are obtained by the po- lycondensation of trimethylamine or triethanolamine with epichlorohydrin [7] and other prefunctionalized monomers [8]. For example, these are MA-40 (Shchekinoazot, Russia), PEC-1000 (Toray Industries, Inc.), FT-30 (Filmtec), etc. The mechanical strength of these membranes is often provided by introducing an inert binder. For example, in the case of quasi-homogeneous membranes of the Neosepta type (Astom, Japan), polyvinyl chloride powder was introduced at the stage of monomer polymerization [9,10]. In the case of heterogeneous membranes such as Ralex AMH MH (MEGA, Czech Republic), MA-41 (Shchekinoazot, Russia), etc., low-pressure polyethylene powder was mixed with ion-exchange resin powder at the stage of hot rolling [11]. However, subsequent studies have shown that electrochemical degradation causes the destruction of polyvinyl chloride and the degradation of the transport properties of anion-exchange membranes (AEMs) during their operation in electrodialysis processes [12,13,14]. Low adhesion of polyethylene and ion exchange resins leads to the formation of macropores, reducing the selectivity of heterogeneous membranes [15,16]. Membranes with new inert fillers and reinforced cloth have appeared on the market. For example, Astom completely replaced the brands of their membranes [17], excluding polyvinyl chloride from the membrane composition [18]. Membranes with novel polymer matrix architectures are becoming commercially available from other manufacturers [19,20,21,22,23]. In particular, Chemjoi CJMAED membranes (Chemjoi Co., Ltd., Hefei, China) [2,24,25] do not contain an inert binder. They are made from functionalized polyolefins or polyvinylidene fluoride (PVDF) using aromatic crosslinkers [26,27]. These membranes are increasingly used in electrodialysis processes for the purification, separation and concentration of various charged species [25,28,29,30,31,32,33]. 

This study is aimed at investigating the behavior of some new anion exchange membranes (ASE, CJMA-3, CJMA-6) in phosphate-containing solutions. Interest in phosphates is due to several reasons. Firstly, phosphates released into the environment from municipal wastewater, animal waste and leachate from solid waste landfills cause eutrophication of water bodies and pose a danger to the environment. The development of low-reagent membrane processes for recovery phosphates from various streams and returning them to production can solve this problem [34,35]. Moreover, electrodialysis has already established itself as a promising method for the extraction and simultaneous concentration of phosphates at the final stage of such circulation schemes [36,37,38,39,40]. Secondly, researchers who are engaged in applied aspects of the development of such electrodialysis processes report low current efficiency [36,41] and relatively high energy consumption [42,43], as well as lower limiting concentrations of phosphates [44,45] which are achieved at electrodialysis processing of phosphate-containing solutions compared to other substances, such as NaCl. For example, the recovery of chlorides and phosphates is 90% and 40%, respectively, if sweet whey demineralization using ED reaches 70% [46]. Selectrodialysis was performed in batch mode using an industrial anaerobic effluent as the feed solution with 2.75 mM H_x_PO_4_^−^, 72 mM NaCl and 21 mM HCO_3_^−^ anions [45]. This solution was pumped through a feed compartment formed by a standart cation exchange membrane (PC-SK) and a standart anion exchange membrane (PC-SA), manufactured by PCA GmbH, Germany. After 60 h, the chloride concentration in the feed stream decreased by almost 100 times, while the phosphate concentration decreased by only 4 times.

One of the explanations for this phenomenon is steric hindrances that arise during the transport of large, highly hydrated phosphoric acid anions through ion-exchange membranes [41,42,47]. In this paper, we show that in addition to steric hindrances, the transfer of phosphates in AEMs is affected by the ion-exchange capacity and composition of fixed groups (strongly basic, weakly basic), as well as by the degree of crosslinking of the polymer matrix. 

## 2. Materials and Methods

### 2.1. Membranes and Solutions

Hefei Chemjoy Polymer Materials Co., Ltd. (Hefei, China) manufactures homogeneous anion exchange CJMA-3 and CJMA-6 membranes. The basis of their ion-exchange matrix is polyvinylidene fluoride (CJMA-3) [26] or polyolefins (CJMA-6). These matrixes are crosslinked with cross-linked agents [27]. Polyethylene terephthalate cloth provides mechanical strength to the membranes. Detailed information about their surface properties as well as diffusion permeability and selectivity, is given in Refs. [48,49]. 

Astom (Yamaguchi, Japan), the manufacturer of the homogeneous Neosepta ASE anion-exchange membrane [17], does not provide information on its chemical structure. At the same time, Chen et al. [18] report that this membrane has a polystyrene matrix cross-linked with divinylbenzene and contains strongly basic amino groups. The reinforcing cloth is made from a mixture of polyethylene and polypropylene. 

Heterogeneous cation-exchange (MK-40) and anion-exchange (MA-41) membranes of Shchekinoazot Ltd. (Shchekino, Russia) are auxiliary in electrochemical measurements. Their characteristics are detailed in [50] and in Supplementary Materials, the SM. Table 1 summarizes some characteristics of the studied membranes.

Salt pretreatment of all membranes preceded the experiments. Then the membranes were equilibrated with the studied solution for at least 8 h. The solutions were prepared from distilled water (electrical resistance of 1.0 ± 0.1 µS cm^−1^, pH 5.6 ± 0.1) and analytical grade crystalline salts (OJSC Vekton, St. Petersburg, Russia) of NaCl or NaH_2_PO_4_; 0.02 M solution with pH 4.4 ± 0.1 and 0.1 M NaOH solution (OJSC Vekton, St. Petersburg, Russia) were used to prepare 0.02 M solutions with pH 6.6 ± 0.1 and 10.0 ± 0.2. The error in determining the concentrations of electrolytes in the solution does not exceed 2%.

Prepared Na_x_H_(3−x)_PO_4_ solutions contain: 1.99·10^−2^ M NaH_2_PO_4_, 3.16·10^−5^ M Na_2_HPO_4_ and 3.35·10^−13^ M Na_3_PO_4_ (pH 4.4 ± 0.1); 1.60·10^−2^ M NaH_2_PO_4_, 4.03·10^−3^ M Na_2_HPO_4_ and 6.77·10^−9^ M Na_3_PO_4_ (pH 6.6 ± 0.1); 1.98·10^−5^ M NaH_2_PO_4_, 1.98·10^−2^ M Na_2_HPO_4_ and 1.33·10^−4^ M Na_3_PO_4_ (pH 10.0 ± 0.2). The component composition of the feed solutions was calculated taking into account the equilibrium dissociation constants of phosphoric acid (see the SM).

### 2.2. Methods

*FTIR spectra* of the membranes were obtained using a Vertex-70 spectrometer (Bruker Optics, Ettlingen, Germany) and OPUS™ 7.5 software.

*The conductivity* of the membranes was determined by a differential method using a clip-cell [51,52] and an immittance meter AKIP 6104 (B + K Precision Taiwan, Inc., New Taipei City, Taiwan) at an alternating current frequency of 1 kHz. In the case of NaCl solutions, the volume fraction of the intergel solution f_2_ and the ion-exchange capacity of the gel phase $\bar{Q}$ were found using the microheterogeneous model [53]. A description of the procedure for determining these parameters is given in the SM. 

**Figure 1:**
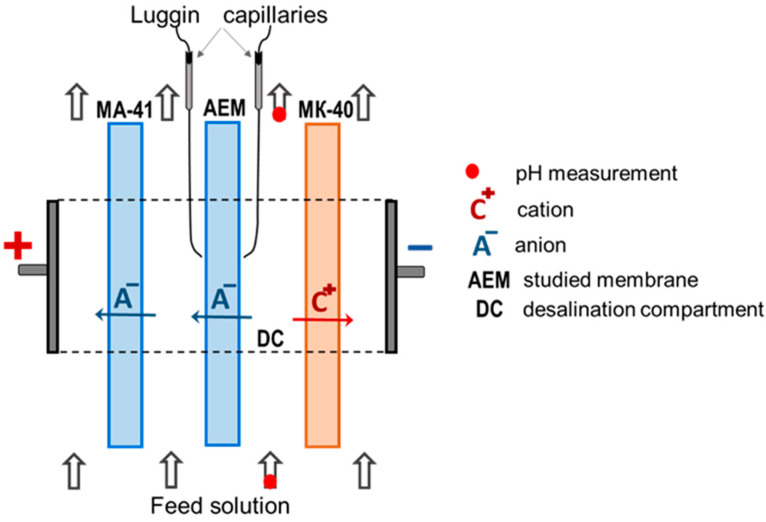
Scheme of the laboratory scale electrodialyzer.

*Current–voltage curves* (CVC) of the studied membranes were obtained using an Autolab PGSTAT-100 electrochemical station Metrohm Autolab B.V. (Kanaalweg, The Netherlands) at the current sweep rate 0.02 mA cm^−2^. The studies were carried out in four-compartment laboratory scale electrodialyzer (Figure 1). The polarizable area of the membrane under study was 2.0 × 2.0 cm^2^. The intermembrane distance was 0.660 ± 0.002 cm. The average linear velocity of the pumped solution was 0.40 ± 0.01 cm·s^−1^. The Luggin capillaries were used to measure the potential drop across the membrane under study. The distance from the capillary tip to the membrane surface was about 0.08 cm. A fresh sample of the membrane was used for each of the tested solutions. A detailed description of the experimental technique and the scheme of the experimental setup is given, for example, in [54] and presented in the SM.

*The pH difference at the outlet and inlet of the desalination compartment* was measured simultaneously with the CVC. The anion-exchange membrane under study and the auxiliary cation-exchange membrane MK-40 formed this compartment. Combined electrodes and an Expert-001 pH meter (Econix-Expert Ltd., Rumyantsevo, Russia) were used for measurements.

The theoretical limiting currents (*i_lim_^Le^*^v^) were calculated using the modified Léveque equation [55]. The derivation of this equation and the procedure for calculating the limiting current for a binary electrolyte (common coion and different counterions), as well as the diffusion coefficients and ion transport numbers that are required for the calculation, are presented in the SM.

The reduced potential drops, Δφ’, were found as Δφ’= Δφ − Δφ_Ω_. Here, Δφ is the potential drop between the Luggin capillaries at i ≠ 0; Δφ_Ω_ = *IR_Ω_*, where *I* is the electric current; *R_Ω_* is the “ohmic” resistance of the membrane under study, including the solution layers between the Luggin capillaries; *R_Ω_* is determined as the slope of the initial section of the CVC at *I*→0.

Batch electrodialysis of 0.03 M Na_x_H_(3−x)_PO_4_ solution with pH 4.4 ± 0.1 was carried out in the same electrodialyzer (Figure 1). The diluate stream contained 100 cm^3^ of feed solution. The other streams contained 1000 cm^3^ of feed solution. The pH of the diluate stream was maintained constant by the continuous addition of 0.1 M NaOH solution. The current density was kept constant, equal to 2.460 ± 0.001 mA cm^−2^. The average linear flow velocity of the pumped solution was 0.40 ± 0.01 cm·s^−1^. Details of the experimental setup and data processing are described in [54] and presented in the SM.

## 3. Results

### 3.1. Chemical Structure of the Membranes

**Figure 2:**
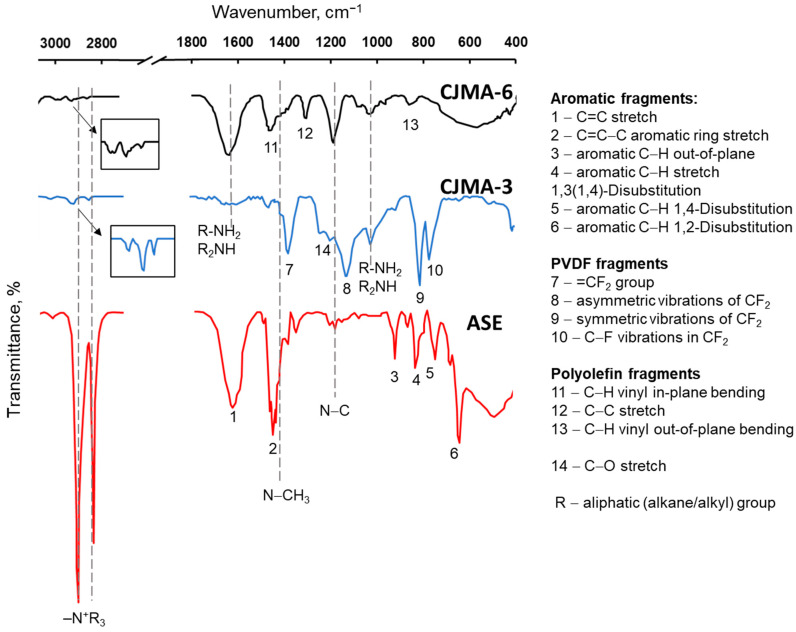
IR spectra of ASE, CJMA-3 and CJMA-6 membranes.

Figure 2 shows the IR spectra, which generally confirm the scattered information [18,26,27,28,48] on the chemical structure of the ASE, CJMA-3 and CJMA-6 membranes.

The IR spectrum of the ASE membrane, as expected, contains peaks relative to the vibrations of the aromatic ring (1610–1440 cm^−1^), in-plane (1000–960 cm^−1^) and out-of-plane aromatic vibrations (950–675 cm^−1^), which explicitly confirms the aromatic nature of their poly(styrene-co-divinylbenzene) matrix [56,57].

The strong absorption bands 1168 cm^−1^, 877 cm^−1^ and 838 cm^−1^ characterize asymmetric and symmetric vibrations of the CF_2_ bond and vibrations of the C-F bond, respectively, in the CJMA-3 membrane. These peaks are characteristic of polyvinylidene fluoride, PVDF [58,59,60]. The IR spectrum of the CJMA-6 membrane shows peaks related to the stretching vibrations of C-C (1303 cm^−1^) and C-H (860 cm^−1^) bonds characteristic of polyolefins. In addition, the IR spectra of the CJMA-3 and CJMA-6 membranes contain a peak at 1403 cm^−1^, related to plane vibrations of the benzene ring, as well as low-intensity bonds in the region 1615–1610 cm^−1^. Taken together, these data support the idea of the presence of a certain amount of aromatic fragments [58,59,60] introduced with crosslinking agents. Peaks 1232 cm^−1^ and 1272 cm^−1^ in the IR spectra of CJMA-3 may indicate a C-O-C bond [61,62,63] in the crosslinker.

As for the fixed groups, the IR spectra of CJMA-6, CJMA-3 and ASE membranes have peaks in the region of 2860–2820 cm^−1^ and at 1426 cm^−1^. They are traditionally attributed to stretching asymmetric and symmetric vibrations and deformation asymmetric and symmetric vibrations of the N–CH_3_ bond or –N^+^R bond [61,64]. These peaks, indicating quaternary ammonium groups [61,65], are more pronounced in the case of ASE and less pronounced in the case of CJMA-3 and CJMA-6 membranes. Apparently, a decrease in the ion-exchange capacity (and the concentration of fixed groups) in the sequence ASE >> CJMA-6 > CJMA-3 causes a decrease in the intensity of the peaks. 

As for weakly basic fixed groups, valence stretching asymmetric and symmetric vibrations in the region of 3500–3300 cm^−1^ are characteristic of primary and secondary amines [61,63,66]. However, a wide band, which is given by free and bound OH groups, overlaps this region [63]. This overlap is often observed in the IR spectra of polymeric ion-exchange membranes [67,68]. Therefore, this region of the spectra is not shown in Figure 2. However, a distinct peak at 1200 cm^−1^ in the CJMA-6 spectrum corresponds to the ν(N–C) stretching vibrations of tertiary amino groups [63,69]. The same IR spectrum has a distinct peak at 1630–1620 cm^−1^ and at 1075 cm^−1^. The authors of some publications [58,61,63,70,71] refer to planar deformation vibrations of the R–NH_2_ or R_2_NH groups. Si- milar but less pronounced peaks occur in the case of the IR spectrum of the CJMA-3 membrane. 

Thus, the ASE membrane contains mainly strong basic quaternary ammonium groups. The same groups, but in smaller amounts present in CJMA-3 and CJMA-6 membranes. Note that the CJMA-6 membrane also contains significant amounts of tertiary, secondary, and primary amines as fixed groups. CJMA-3 and ASE membranes also contain weakly basic fixed groups but in much smaller amounts. In the case of ASE, a peak at 1200 cm^−1^ is observed. According to some reports [72], reinforcing fibers from a mixture of polyethylene and polypropylene are functionalized with tertiary amines to increase adhesion with the ion-exchange material.

### 3.2. Conductivity

**Figure 3:**
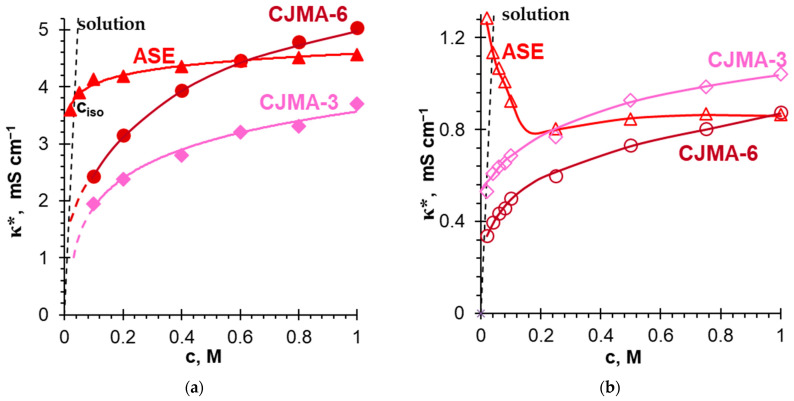
Concentration dependences of the conductivity of ASE, CJMA-3, and CJMA-6 membranes in NaCl solutions with pH 5.5 ± 0.01 (**a**) and in NaH_2_PO_4_ solutions with pH 4.4 ± 0.01 (**b**).

Figure 3 shows the concentration dependences of the conductivity of the studied membranes in solutions of NaCl (pH 5.5 ± 0.1) and Na_x_H_(3−x)_PO_4_ (pH 4.4 ± 0.1). The results of processing these dependencies using the microheterogeneous model [53] are presented in Table 1 for NaCl solutions. A detailed description of the microheterogeneous model and its application is given in the SM.

Within the framework of the microheterogeneous model, any ion-exchange membrane with a pore size of more than 1–3 nm consists of two phases. The gel phase (*1*) includes a polymer matrix with fixed groups whose electric charge is counterbalanced by a charged solution containing mobile counterions and, to a lesser extent, co-ions. This phase includes only micropores less than 3 nm in size. The central parts of the meso- and macropores are filled with an electrically neutral solution from another phase called “the intergel solution” (*2*). The concentration and composition of this phase are identical to the bathing (external) solution. The sum of the volume fractions of both phases is 1 ($f_{1}+f_{2}=1$). The ion-exchange capacity of the gel phase can be easily determined from the relationship $\bar{Q}=Q/f_{1}$, where Q is the ion-exchange capacity of the membrane. Since *f*_1_ is rather close to 1, $\bar{Q}$ is slightly higher than Q.

The conductivities of the membrane ($\kappa^{*}$), gel phase ($\bar{\kappa }$) and intergel solution (κ) are related by the equation:

(1) $$\kappa^{*}={\bar{\kappa }}^{f_{1}}\kappa^{f_{2}}$$

This equation is valid in the range of equivalent (eq L^−1^) electrolyte concentrations from 0.1C*iso* to 10*C_iso_*, if the parameter α is not too large, $\left|\alpha \right|\leq 0.2.$ Here $C=\left|z_{1}\right|c_{1}=\left|z_{A}\right|c_{A}$; *c_i_* (*i* = 1, A) (mol L^−1^) is the molar ion concentration. Subscripts *1* and *A* denote the counterion and coion. *C_iso_* is the isoconductance concentration at which the conductivities of the membrane, gel phase and bathing solution are the same. α is the parameter reflecting the connection of the elements of different phases. If the elements of the gel and intergel phases are connected in series, α is equal to −1; if these elements are connected in parallel, α is equal to 1.

If the concentrations are close to *C_iso_*, the conductivity of the gel phase can be presented approximately by the equation:

(2) $$\bar{\kappa }=\frac{z_{1}{\bar{D}}_{1}\bar{Q}F^{2}}{RT}$$

Here, $\bar{D}$ is the diffusion coefficient of the counterion (*1*); *F*, *R* and *T* are the Faraday constant, absolute temperature and gas constant, respectively.

The shape of concentration dependences of the conductivity of the membranes under study in NaCl solutions (Figure 3a) is in good agreement with similar results obtained for different homogeneous membranes by researchers [48,50,73,74,75,76]. 

These dependencies (Figure 3a) can be interpreted using the microheterogeneous model [6,50,53]. At electrolyte concentrations close to C*_iso_* (C*_iso_* is in the range of 0.01–0.04 M), the membrane conductivity is equal to the conductivity of the gel phase, $\bar{\kappa }$. The value of $\bar{\kappa }$ is proportional to the concentration of counterions, which is practically equal to the concentration of fixed groups, $\bar{Q}$ (see Equation (2)), due to the Donnan exclusion of coions. The higher $\bar{Q}$, the higher $\bar{\kappa }$, and, accordingly, the higher $\kappa^{*}$. Indeed, the conductivity of the studied membranes in dilute solutions increases in the same sequence as their ion-exchange capacity of the gel phase: CJMA-3 < CJMA-6 < ASE (Figure 3a, Table 1). In the region of decimolar and more concentrated solutions, the main contribution to the values of $\kappa^{*}$ is made by the conductivity of the intergel solution. The higher the *f*_2_ value (Table 1), the greater the slope of the curve in Figure 3a. 

Note that the basic version of the microheterogeneous model [53] takes into account only electrostatic interactions between fixed groups and counterions. It does not consider the possibility of changing the ionic composition of the gel phase in comparison with the electrically neutral solution filling the intergel spaces. However, such phenomena just take place in the case of phosphate-containing solutions, as was shown in previous studies [77]. Getting into the gel phase of the membrane, singly charged H_2_PO_4_^−^ anions are partially converted into doubly charged HPO_4_^2−^ anions. The reason for this transformation is the participation of H_2_PO_4_^−^ in protonation-deprotonation reactions with water, which result in the formation of HPO_4_^2−^ anions and protons. The latter are coions and are excluded from the membrane due to the Donnan effect. According to Equation (2), doubling the electric charge of counterions can lead to an increase in $\bar{\kappa }$ (if the diffusion coefficient of HPO_4_^2−^ in AEM is not much lower than that of H_2_PO_4_^−^). 

The Donnan exclusion increases with a dilution of the bathing (external) solution [6]. This leads to an increase in the pH of the AEM internal solution and, consequently, to an increase in the equivalent fraction of doubly charged HPO_4_^2−^ ions in the membrane and to an increase in its conductivity. Thus, the conductivity of AEMs can increase upon dilution of the NaH_2_PO_4_ solution (such as in the case of the ASE membrane, Figure 3b). This increase is theoretically substantiated and experimentally proven not only for phosphates but also for solutions of salts of organic polybasic acids if the membrane contains mainly quaternary ammonium groups [77]. In the case of electrolyte solutions that do not participate in protonation–deprotonation reactions (NaCl), the electrical conductivity decreases when the solution is diluted. CJMA-3 and CJMA-6 membranes have a rather low ion-exchange capacity of the gel phase (Table 1). Accordingly, the Donnan exclusion of coions (protons) in the gel phase of these membranes is reduced compared to the ASE membrane. Therefore, the enrichment of the membrane with doubly charged anions of phosphoric acid is less pronounced. Thus, it does not lead to an increase in conductivity. However, this effect manifests itself in an apparent decrease in the value of *f*_2_ by 9 ± 1% compared to the NaCl solution (see the SM).

The diffusion coefficients of anions in solution are equal to 2.03 × 10^−5^ (Cl^−^), 0.959 × 10^−5^ (H_2_PO_4_^−^) and 0.799 × 10^−5^ (HPO_4_^2−^) (all in cm·s^−1^) [78]. Let us assume that the ratio of the mobilities of these anions in the gel phase of the membrane remains the same as in the solution. In accordance with Equation (2), the conductivity of the membranes under study should decrease by a factor of 2.1 or 1.3 compared to the conductivity in NaCl solution if the gel phase contains 100% H_2_PO_4_^−^ anions or 100% HPO_4_^2−^ anions, respectively. This prediction is valid for solutions with NaH_2_PO_4_ concentrations close to C*_iso_* (0.04 M) when the conductivities of the membrane and the gel phase are approximately the same. However, only the CJMA-3 membrane shows a decrease in conductivity in the expected range (Table 2). The decrease in conductivity of ASE and CJMC-6 is more significant (Table 2).

The low *f*_2_ value of the ASE membrane (Table 1) indicates a high degree of crosslinking of its polymer matrix (which reduces the presence of large pores). Apparently, large and highly hydrated phosphoric acid anions encounter steric hindrance in this matrix during transport. On the contrary, the CJMA-6 membrane has the highest *f*_2_ value (Table 1), which indicates weak crosslinking and the presence of rather large pores in the polymer matrix. In this case, steric hindrance can hardly be the main reason for the decrease in the conductivity of CJMA-6 in NaH_2_PO_4_ solution compared to NaCl. Probably, the weakly basic amino groups of the CJMA-6 membrane (Section 3.1) are the main reason for the decrease in its conductivity in phosphate-containing solutions. Really, the equilibrium constants for the protonation–deprotonation reactions are of the order of 10^−5^–10^−3^ mmol cm^−3^ for the primary and secondary amino groups and 10^−8^–10^−7^ mmol cm^−3^ for the tertiary amino groups [7,79,80]. This means that weakly basic amino groups become fully protonated at pH < 3 and fully deprotonated at pH > 8. Therefore, the ion-exchange capacity of AEMs with weakly basic fixed amino groups reaches a maximum at pH < 3, decreases with increasing pH, and reaches a minimum at pH > 8 [80]. According to Equation (2), a decrease in the ion-exchange capacity of AEM leads to a decrease in their conductivity compared to membranes with strongly basic ammonium groups [81,82]. In our case, this phenomenon causes the sharpest decrease in the conductivity of CJMA-6 in dilute NaCl solutions compared to ASE and CJMA-3 membranes (Figure 3a).

Note the pH of the AEMs internal solution is 3 or more units higher compared to the bathing (external) NaH_2_PO_4_ solution with a pH of 4.4 ± 0.1. This fact was proved experimentally by the method of color indication [54]. Therefore, CJMA-6 weakly basic amino groups (Section 3.1) are partially deprotonated and do not participate in the transfer of counterions. However, a decrease in CJMA-6 conductivity compared to ASE and CJMA-3 membranes occurs not only in dilute but also in concentrated NaH_2_PO_4_ solutions (Figure 3b). This behavior of the CJMA-6 membrane makes us think about another mechanism of inhibition of phosphate transport. We will discuss it in Section 3.4.

### 3.3. Current-Voltage Curves

*NaCl solutions*

**Figure 4:**
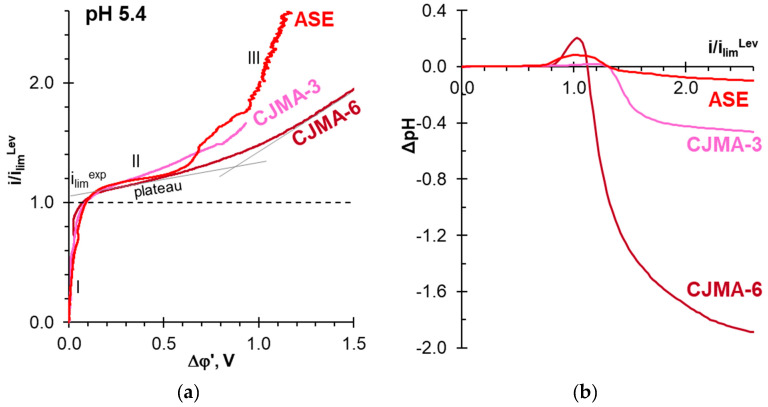
Current-voltage curves of the studied membranes in 0.02 M NaCl solution (**a**) and the difference between the pH of the solution at the outlet and at the inlet of the desalination compartment (**b**) vs. the current density. The current density is normalized to the limiting current density calculated using the Leveque Equation (S27).

Current-voltage curves of ASE, CJMA-3 and CJMA-6 membranes in 0.02 M NaCl solution are shown in Figure 4a. Figure 4b presents the dependence of the difference between the pH of the solution at the outlet and at the inlet of the desalination compartment upon the current density obtained simultaneously with the CVC. Recall that the studied anion-exchange membrane and the auxiliary cation-exchange membrane MK-40 form the desalination compartment of the electrodialysis cell (Figure 1). The current density is normalized to the theoretical limiting current density calculated using the Leveque equation (see the SM, Equation (S27)).

The shape of these CVCs is typical for the curves obtained for membranes in a solution of electrolytes that do not participate in protonation–deprotonation reactions [83,84]. The initial section *I* is followed by a section of a sloping plateau *II*. The intersection point of the tangents to these sections gives the value of the experimental limiting current, *i_lim_^exp^*, which is close to the theoretical limiting current, *i_lim_^Lev^*. Electroconvection, which develops as electroosmosis of the first kind [71,72,73], causes an increase in *i_lim_^exp^* over *i_lim_^Lev^* by 5–7%. Increasing the ion-exchange capacity (Table 1) contributes to an increase in *i_lim_^ex^*/*i_lim_^Lev^* value. Really, the greater the concentration of fixed groups, the greater the magnitude of the electric charge on the membrane surface, which controls the driving force in electroosmosis [85]. In overlimiting mode (*i > i_lim_^Lev^*), water splitting occurs at the AEM/diluted solution boundary, which leads to acidification of the desalted solution. Water splitting is minimal in the case of ASE due to the low catalytic activity of strong basic fixed groups (Figure 2) towards water splitting [86,87]. In addition, this membrane has a relatively smooth surface [88], which does not contribute to the formation of stagnant zones. The CJMA-3 membrane also contains predominantly strongly basic fixed groups (Section 3.1). However, its surface with “valleys” and “hills” is very undulating [88]. A significant decrease in the concentration of NaCl in the stagnant zones of the “valleys” enhances water splitting (and acidification of the solution) compared to ASE (Section 3.1). The most significant acidification of the solution occurs in the case of the CJMA-6 membrane, which contains a significant proportion of weakly basic fixed amino groups (Section 3.1). These groups are actively involved in proton transfer reactions, contributing to water splitting [86,87,89].

The “plateau length” of the CVC is a potential drop between the point that determines *i*_lim_^exp^ and the point of intersection of the tangents to sections II and III. The “plateau length” increases in the sequence ASE < CJMA-3 < CJMA-6. As a rule, the “plateau length” corresponds to the threshold potential drop at which unsteady Rubinstein–Zaltzman electroconvection develops. The space charge at the AEM surface governs electroconvection [90,91]. Protons, which are products of water splitting, reduce the space charge density and suppress electroconvection [92]. Therefore, the value of the threshold potential drop (“plateau length”) increases with the intensification of water splitting.

In the case of ASE, insignificant water splitting practically does not hinder the development of electroconvection. Large electroconvective vortices deliver a more concentrated solution from the bulk, increasing the conductivity of the membrane system under study. As a result, the slope of the CVC section III of this membrane is significantly greater than in the case of CJMA-3 and CJMA-6 membranes. Oscillations of the potential drop in this section of the ASE current-voltage curve indicate the development of unsteady electroconvection. On the contrary, in the case of CJMA-6, water splitting leads to a decrease in the size of electroconvective vortices and a slowdown in their rotation rate [92]; as a result, the slope of the CVC section III for this membrane is quite low. The case of CJMA-3 membrane is an intermediary in terms of both the rate of H^+^/OH^−^ generation and the intensity of electroconvection.

*Na_x_H_(3−x)_PO_4_ solutions*

**Figure 5:**
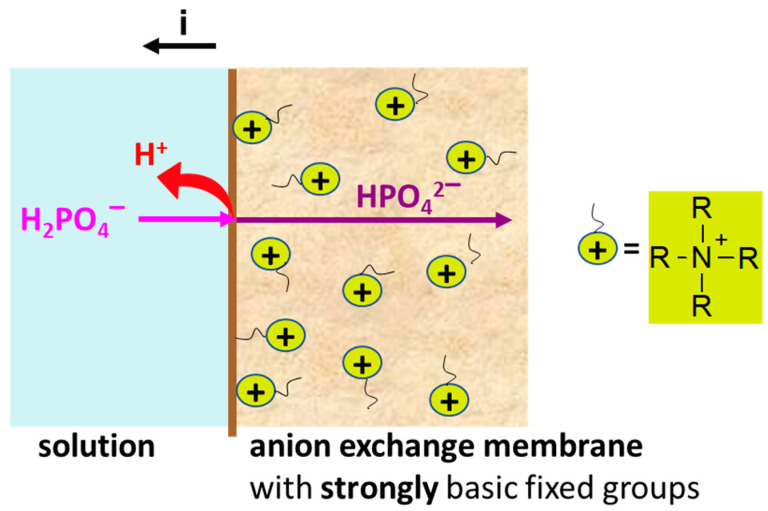
Schematic representation of the influence of the “acid dissociation” phenomenon on the generation of protons and the transfer of phosphoric acid species in an anion exchange membrane containing only strongly basic fixed groups.

In the case of Na_x_H_(3−x)_PO_4_ solutions, H^+^/OH^−^ ions are generated by two mechanisms. Water splitting does not differ from that in solutions of strong electrolytes, i.e., it becomes essential at *i > i_lim_^Lev^* and occurs with the participation of the fixed groups [86,87,89,93]. One more mechanism, which we call “acid dissociation” for brevity [54,93], takes place if the solution contains species capable of dissociating with the release of protons (Figure 5). Anions of phosphoric and other organic or inorganic polybasic acids are such species. As mentioned in Section 2.1, Na_x_H_(3−x)_PO_4_ solution with pH 4.4 ± 0.1 contains 99% HPO_4_^−^ anions. These anions, getting into AEM, dissociate with the formation of protons and doubly charged HPO_4_^2−^ anions. The electric field promotes the electromigration of HPO_4_^2−^ anions through AEM into the concentration compartment. Protons enter the depleted solution of the desalination compartment due to the Donnan exclusion of coions from AEM. This phenomenon takes place at any current density, including *i < i_lim_^Lev^*.

**Figure 6:**
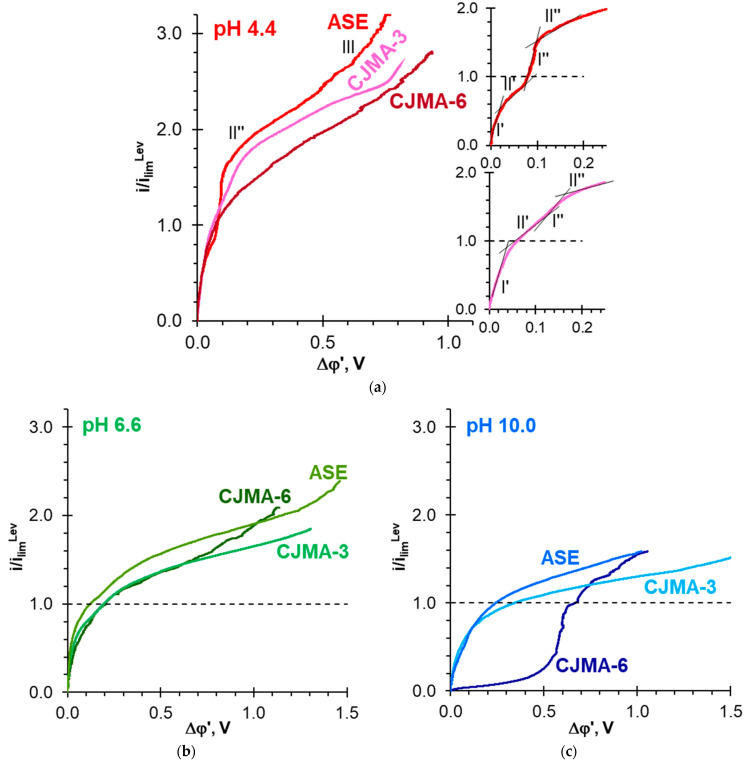
Current-voltage curves of ASE, CJMA-3, and CJMA-6 membranes in 0.02 M Na_x_H_(3−x)_PO_4_ solutions with pH 4.4 ± 0.1 (**a**), 6.6 ± 0.1 (**b**) and 10.0 ± 0.2 (**c**). The current density is normalized to the limiting current calculated using the modified Leveque Equation (S35).

**Figure 7:**
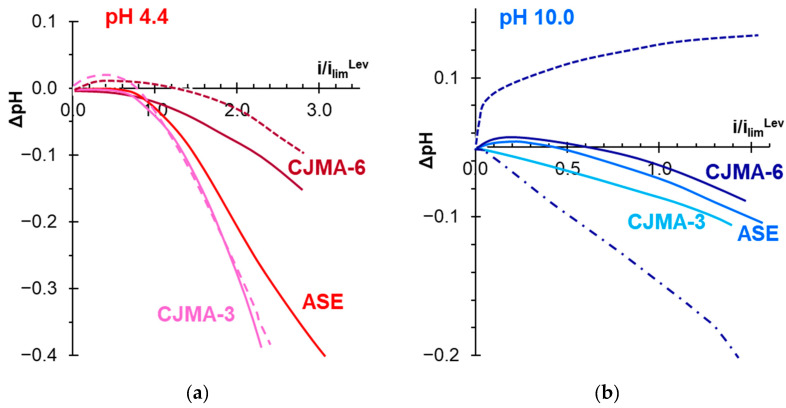
The difference between the pH of the solution at the outlet and at the inlet of the desalination compartment vs. current density normalized to the theoretical limiting current. The data were obtained simultaneously with the current–voltage curves of membranes under study in 0.02 M Na_x_H_(3−x)_PO_4_ solutions with pH 4.4 ± 0.1 (**a**) and 10.0 ± 0.2 (**b**). The solid lines correspond to a preliminary exposition of the studied membranes under current for 1 h. The dashed and dash-dotted lines correspond to the exposition under current for 0 and 5 h, respectively.

The occurrence of two sloping plateaus on the CVCs of ASE and CJMA-3 membranes (Figure 6a) is a consequence of the “acid dissociation” mechanism [54,93]. The first plateau II′ in the *i_lim_^Lev^* vicinity has the same nature as in the case of NaCl solutions. It corresponds to a sharp increase in the resistance of the depleted solution at the AEM surface caused by electrodiffusion limitation in the delivery of electrolyte (NaHPO_4_) from the bulk to the interface. Dilution of the solution near AEM stimulates an increase in the Donnan exclusion of H^+^ coions from the membrane. The increase in the conductivity of the depleted solution due to its enrichment with protons and the doubling of the electrical charge of the counterions that are transported through the AEM causes an increase in the current density between plateaus II′ and II″. Plateau II″ corresponds to the depletion of the proton source during the transport of predominantly doubly charged HPO_4_^2−^anions through the AEM. The ASE membrane has a higher ion-exchange capacity compared to the CJMA-3 membrane (Table 1). This results in a stronger Donnan exclusion of protons in ASE compared to CJMA-3 due to a greater electrostatic repulsion force [7]. Accordingly, the value of the current density corresponding to plateau II″ is higher in the case of ASE compared to CJMA-3. At the same time, plateau II′ on the CJMA-3 membrane CVC is more diffuse. At any *i/i_lim_^Lev^* value, the “acid dissociation” mechanism provides relatively strong acidification of the solution at the outlet of the desalination compartment in the case of ASE and CJMA-3 membranes. Moreover, the duration of exposure of membranes under current has little effect on this acidification (Figure 7a). As the current density increases, the membranes become more and more enriched in doubly charged anions. This enrichment slows down the rate of release of protons into the depleted solution. The deficiency of electric charge carriers (HPO_4_^−^, H^+^) in this solution stimulates the generation of protons and hydroxyl ions by the water splitting mechanism at current densities corresponding to plateau II″.

Both “acid dissociation” and “water splitting” mechanisms occur in parallel at overlimiting currents (section III of the CVC) [54]. In addition, at approximately the same current densities, rather large electroconvective vortices appear in the depleted solution near the ASE surface. The presence of these vortices is evidenced by small oscillations of the potential drop observed on the CVC of the ASE membrane at *i* > 2.5 *i_lim_^Lev^*. As in the case of NaCl solutions, the development of electroconvection contributes to an increase in the mass transfer rate to the AEM surface [92]. Consequently, the slope of the ASE current–voltage curve becomes greater at Δφ′ > 1.3 V (Figure 6a).

Na_x_H_(3−x)_PO_4_ solution with pH 10.0 ± 0.2 contains about 99% H_2_PO_4_^−^ and 1% PO_4_^3−^. The pseudo-unimolecular rate constant of the rate-limiting step of the HPO_4_^2−^ ↔ PO_4_^3−^ + H^+^ reaction is very low, 5·10^−3^ s^−1^ [54]. For the conversion of H_2_PO_4_^−^ anions into PO_4_^3−^ anions due to the “acid dissociation” mechanism, a relatively high potential difference is needed. Therefore, this mechanism has practically no effect on the shape of ASE and CJMA-3 current-voltage curves. This shape remains approximately the same as in the case of NaCl solutions (Figure 4a and Figure 6c). Acidification of the desalted solution at *i < i_lim_^Lev^* does not exceed the experimental error (Figure 7b), but becomes more significant at *i > i_lim_^Lev^*.

The CVC of ASE and CJMA-3 membranes obtained in Na_x_H_(3−x)_PO_4_ solution with pH 6.6 ± 0.1, which contains about 20% HPO_4_^2−^ and 80% H_2_PO_4_^−^, have an intermediate shape (Figure 6b). Since this solution has buffer properties, “acid dissociation” and water-splitting mechanisms have practically no effect on the pH of the desalted solution.

Thus, the CVC shape and the ability of ASE and CJMA-3 membranes to acidify the desalted solution are similar to the results we obtained earlier for other anion-exchange membranes (AMX, AMX-sb and AX by Astom, Japan) [54,92,93], which mainly contain quaternary ammonium fixed groups.

The CJMA-6 membrane behaves differently. In Na_x_H_(3−x)_PO_4_ solutions with pH 4.4 ± 0.1 or 6.6 ± 0.2, the CVCs have only one plateau (Figure 6a,b), which is observed at lower current densities than in the case of the CJMA-3 membrane. Note that the CJMA-6 membrane has a higher ion-exchange capacity compared to the CJMA-3 membrane. Therefore, in the case of CJMA-6, one would expect a more significant increase in the *i_lim_^exp^* (plateau II″) caused by the “acid dissociation” mechanism. The desalted solution acidification is less significant than in the case of ASE and CJMA-3 membranes (Figure 7a). Moreover, the degree of acidification increases with increasing duration of exposure of CJMA-6 membrane undercurrent.

In Na_x_H_(3−x)_PO_4_ solutions with pH 10.0 ± 0.2, the CJMA-6 current-voltage curve has a shape (Figure 6c) characteristic of bipolar membranes [94]. In the case of a “fresh” membrane (i.e., the membrane that has not previously been exposed to an electric field), the desalted solution is slightly alkalized (Figure 7b). After operating in an electric field for several hours, the desalted solution becomes more and more acidic (Figure 7b). In 5 h, this acidification becomes more significant compared to ASE and CJMA-3 membranes.

Specific interactions of weakly basic fixed groups with proton-containing phosphoric acid anions appear to be the cause of the abnormal behavior of CJMA-6.

According to Refs. [95,96,97,98], specific interactions between primary or secondary amines and acid residues of oxoacids play a key role in the self-organization of many native supramolecular structures. Probably, such interactions provide the high sorption capacity of anion-exchange resins with fixed phosphonate groups with respect to amino acids [99] and create a special architecture of the ion-exchange membrane surface upon layer-by-layer modification with polyallylamine and sulfonated polystyrene [100,101,102]. Analytical sensors based on these specific interactions are being actively developed [86,87,88], including phosphate-responsive nanofluidic diode [103] and polyamine-functionalized graphene field-effect transistors [101].

The electrostatic/hydrogen bond switching model has been developed to explain the self-organization of native structures [93,94]. It was also used to predict specific interactions between phosphates in solution and the surfaces of analytical sensors functionalized with primary amines [103,104,105,106]. According to the model, bound species from weakly basic amines and anions of oxoacids are formed due to proton transfer reactions, such as the following:

H_2_O ↔ OH^−^ + H^+^ (3)

H_2_PO_4_^−^ ↔ HPO_4_^2−^ + H^+^ (4)

R–N^+^H_3_ ↔ R–NH_2_ + H^+^ (5)

R–N^+^H_3_ + H_2_PO_4_^−^ ↔ R–[(NH_3_)H_2_PO_4_]^0^ (6)

R–N^+^H_3_ + HPO_4_^2−^ ↔ R–[(NH_3_)HPO_4_]^−^ (7)

Electrostatic interactions between species with positive and negative electric charges are enhanced by hydrogen bonds between the oxygen atoms of phosphates and the hydrogen atoms of amines. These hydrogen bonds facilitate the deprotonation of singly charged anions H_2_PO_4_^−^ → HPO_4_^2−^ + H^+^. The binding constant for divalent HPO_4_^2−^ anion is one order of magnitude higher than the binding constant for divalent anions of sulfuric and oxalate acids [102]. Laucirica et al. [104] have found the values of ζ-potential of the surface of nanoparticles functionalized with –NH_2_ groups in phosphate-containing solutions. Using these values and equilibrium constants of reactions (3)–(7), they estimated the mole fraction of the bound species on this surface. For example, in 0.02 M phosphate solution the mole fraction of –[(NH_3_)H_2_PO_4_]^0^ and –[(NH_3_)HPO_4_]^−^ species exceeds 0.3 when pH is in the range from 4 to 10. In more acidic solutions, positively charged protonated amines (e.g, –N^+^H_3_) predominate. In more alkaline solutions, deprotonated amines (e.g., –NH_2_) that have no electrical charge predominate. The species –[(NH_3_)HPO_4_]^−^, which are detailed by Equation (8), impart a negative charge to the surface of functionalized nanoparticles at pH > 6. We believe that Equation (8) is more suitable for describing the mechanism of bound species formation in an anion-exchange membrane:

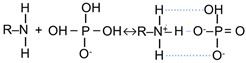
(8)Here, a thin solid line marks the electrostatic interactions; a dotted line marks the hydrogen bonds.

As we discussed in Section 3.2, in the case of Na_x_H_(3−x)_PO_4_ solutions with pH 4.4 ± 0.1, the internal AEM solution has pH values that are shifted to the alkaline range by 3 and more units [54]. Under these conditions, primary and secondary aliphatic amines, which are characterized by equilibrium dissociation constants of 10^−3^–10^−5^ [7,79,80], are partially deprotonated in the volume of AEMs and are protonated on their surface. In the case of Na_x_H_(3−x)_PO_4_ solutions with pH 6.6 ± 0.1 and 10.0 ± 0.2, the weakly basic fixed amino groups of CJMA-6 are uniquely deprotonated in the volume and mostly deprotonated on the membrane surface.

**Figure 8:**
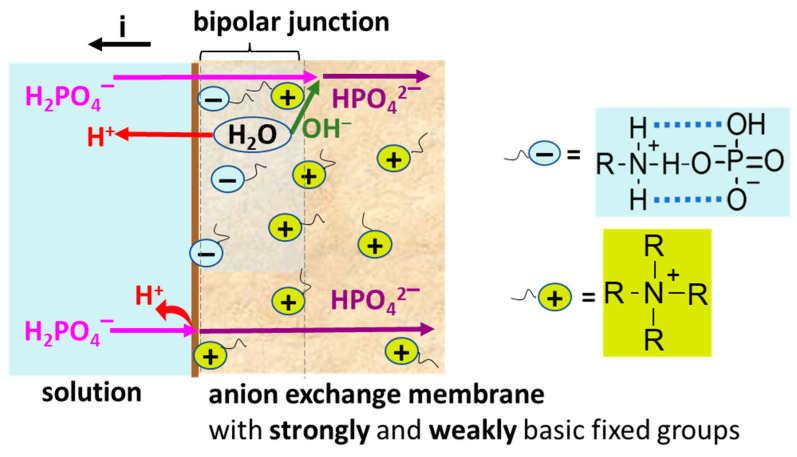
Schematic representation of the generation of protons and the transfer of phosphoric acid species in an anion exchange membrane containing weakly basic and strongly basic fixed groups.

The general idea is that protic anions of phosphoric acids are involved in the formation of bound species of the types (6) and (8). Therefore, weakly basic amino groups acquire a neutral or negative electrical charge. The formation of these bound species results in a decrease in CJMA-6 membrane ion-exchange capacity. It is important to take into account that in conditions of electric current flow, the transfer of H_2_PO_4_^−^ anions occurs from the depleted solution into the membrane. A decrease in the ion-exchange capacity of the near-surface region of the membrane reduces the Donnan effect of proton exclusion, which underlies the generation of protons by the “acid dissociation” mechanism. The less the proton exclusion from the membrane, the less the rate of proton release into the depleted solution (Figure 8).

Indeed, as Figure 7a,b show, the change in pH of the solution passing through the desalination compartment is positive for a fresh CJMA-6 membrane, while it is negative for ASE and CJMA-3 membranes, which (almost) do not contain weakly basic functional groups. However, as these figures show, in the case of CJMA-6 membrane exposed under electric current for more than 1 h, the rate of proton generation is significantly increased and became greater than in the case of ASE and CJMA-3 membranes (at pH 10.0 ± 0.2). Apparently, the reason is the formation of a bipolar junction in the near-surface membrane region of the CJMA-6 membrane (Figure 8). Under conditions of electric current and high pH of the feed solution, more and more weakly basic groups adjacent to the membrane surface transform into negatively charged bound species according to Equation (7) or similar reactions [103,104,105,106]. This process takes some time (apparently a few hours); it can lead to the formation of a negatively charged layer near the surface. The positively charged layer could be preferentially formed by quaternary ammonium groups, which are also present in this membrane. These groups have only weak interactions similar to that presented by Equations (6) and (7) [95,107]. Really, ΔpH vs. *i/i_lim_^Lev^* dependance for the CJMA-3 does not depend on the time of membrane exposure under electric current (Figure 7a).

The bipolar junction at the membrane surface of the CJMA-6 membrane, which apparently is formed in a phosphate solution with pH 10 ± 0.2, behaves like a bipolar ion-exchange membrane. When an external electric field is applied, cations and anions leave the bipolar interface, which leads to the appearance of a depletion layer between the negatively and positively charged regions of the membrane. With increasing electric current, there is a very small linear section of the CVC (Figure 6c), which is followed by an inclined plateau. The plateau corresponds to a limiting current caused by the saturation of diffusion ions delivery to the depletion layer. Then (~0.5 V), a fast increase in electric current with a slight increase in the potential drop appears (Figure 6c), which is caused by the intensive generation of H^+^ and OH^−^ ions in the bipolar junction like in a bipolar membrane. Kooijman et al. [95,108] showed using ^31^P-NMR that the interaction between amines and phosphates promotes both a higher protonation degree of the amines and a higher dissociation degree of the phosphate anions. Apparently, the electric field contributes to a shift in the chemical equilibrium of these reactions due to the removal of the protons into the bathing solution and the phosphate anions into the AEM bulk. The validity of this mechanism is confirmed by thse very high rate of proton generation at the AEM/depleted solution interface in the case of NaH_2_PO_4_ solution [93] compared to NaCl solution.

Thus, weakly basic fixed amino groups of an anion-exchange membrane radically change their characteristics in an electric field in the presence of phosphate anions. The formation of the bond species consisting of phosphates and weakly basic amino groups causes these differences, most likely.

### 3.4. Batch Electrodialysis of Na_x_H_(3−x)_PO_4_ Solution

Batch electrodialysis of 0.03 M Na_x_H_(3−x)_PO_4_ solution with pH 4.4 ± 0.1 (99% NaH_2_PO_4_) was carried out at a current density of 2.460 ± 0.01 mA cm^−2^. Recall that the pH in the diluate stream was maintained constant by the continuous addition of 0.1 M NaOH solution. Other details of the experiment are given in Section 2.2 and in the SM.

**Figure 9:**
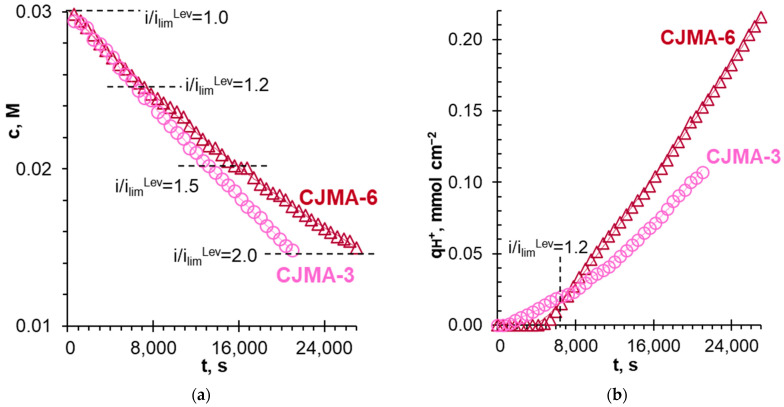
NaH_2_PO_4_ concentration in the diluate stream (**a**) and the number of protons generated by anion-exchange membranes (**b**) vs. batch electrodialysis duration. The data were obtained at a constant current density of 2.46 ± 0.01 mA cm^−2^. The dashed lines show the *i/i_lim_^Lev^* ratios, which correspond to the concentration reached in the diluate stream.

**Figure 10:**
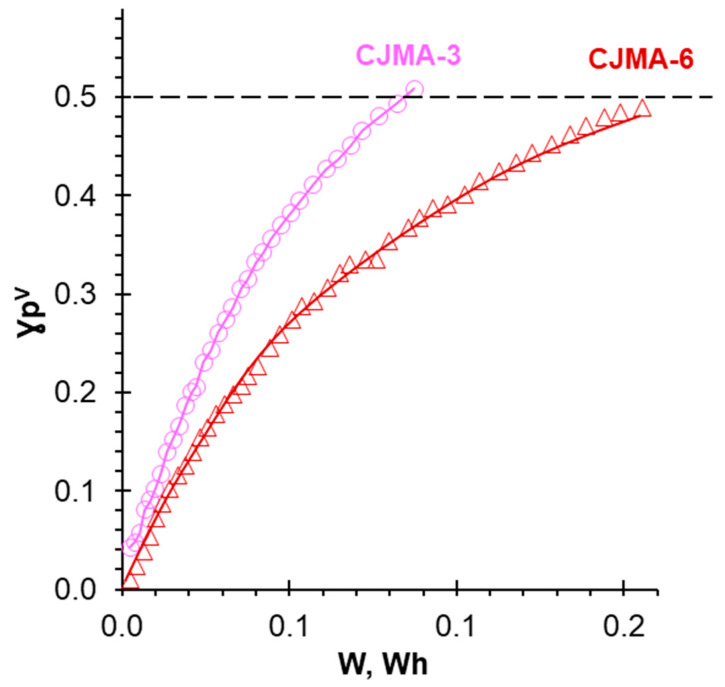
The degree of recovery of pentavalent phosphorus from the diluate stream vs. energy consumption during electrodialysis of an initially 0.03 M NaH_2_PO_4_ solution. CJMA-3 and MK-40 or CJMA-6 and MK-40 membranes form the desalination compartments.

Figure 9 shows the concentration of NaH_2_PO_4_ in the diluate stream and the number of moles of protons generated by the studied AEM, q_H+_, depending on the electrodialysis duration. The value of q_H+_ (in mmol cm^−2^) is found taking into account the concentration and volume of alkali added to the diluate stream to maintain there a constant pH. The dashed lines show the current NaH_2_PO_4_ concentrations in the diluate stream, which correspond to some given *i/i_lim_^Lev^* ratios. Figure 10 represents the recovery degree of pentavalent phosphorus (P^V^), $\gamma_{P^{V}}$, from the diluate stream as a function of the energy consumption; $\gamma_{P^{V}}=(c_{0}-c_{t})/c_{0}$, where $c_{0}$ and $c_{t}$ are the initial and current at time *t* concentrations of P^V^ in the diluate stream, respectively. As Figure 9 shows, the twofold desalination of the NaH_2_PO_4_ solution is 1.3 times faster and requires 1.9 times less energy consumption when using the CJMA-3 membrane compared to CJMA-6.

Note that a decrease in the concentration of the desalted solution is accompanied by a decrease in the limiting current density (*i_lim_^Lev^*), hence, an increase in the *i/i_lim_^Lev^* ratio. At the beginning of electrodialysis, this ratio is 1.0 and reaches a value of 2.0 at the end of the experiment (Figure 9a). Note that for both membranes, desalination rates are equal (within the measurement error) until the concentration of NaH_2_PO_4_ in the diluate stream decreases to 0.025 M. This concentration corresponds to *i/i_lim_^Lev^* ≈ 1.2. At the initial stage of desalination (up to 6000 s), there is an insignificant generation of protons by the CJMA-3 membrane (Figure 9b). At the same time, proton generation by the CJMA-6 membrane is absent within the measurement error (Figure 9b).

As already discussed in Section 3.3, apparently, the formation of the bound species involving weakly basic fixed groups and protic phosphoric acid anions reduces the proton generation by this membrane. A noticeable decrease in the rate of NaH_2_PO_4_ solution desalting takes place in the case of CJMA-6 compared to CJMA-3 at the next stages of desalting, *t* > 6000 s (Figure 9a). This decrease is caused by more intense proton generation (and, accordingly, by more intense transport of doubly charged HPO_4_^2−^ anions across the membrane occurring after exposure of the CJMA-6 membrane to the electric current. The reason seems to be the specific interactions of weakly basic fixed amino groups with phosphoric acid species at the CJMA-6/depleted solution interface already discussed in Section 3.3.

## 4. Conclusions

The performed study shows that the ASE membrane has an aromatic ion exchange matrix and mainly contains quaternary amines as fixed groups. The CJMA-3 and CJMA-6 membranes have aliphatic matrices based on polyvinylidene fluoride and polyolefin, respectively. In addition, the matrices of both CJMA membranes contain aromatic fragments introduced at the stage of crosslinking of aliphatic chains. Quaternary amines are fixed groups of both membranes. However, CJMA-6 contains some weakly basic amines. The volume fraction of intergel spaces filled with an electrically neutral solution increases in the sequence ASE << CJMA-3 < CJMA-6, which indirectly indicates a more significant crosslinking of the ASE polymer matrix compared to aliphatic CJMA membranes.

The conductivity of the membranes correlates with their ion-exchange capacity (the concentration of fixed groups in the membrane) in relatively dilute (<0.6 M) NaCl solutions: ASE > CJMA-3 > CJMA-6. In a dilute solution of NaH_2_PO_4_ with pH 4.4 ± 0.1 (at the isoconductance concentration equal to 0.04 M), the membrane conductivity decreases by a factor of 3.3 ± 0.1 (ASE), 1.8 ± 0.1 (CJMA-3) and 4.5 ± 0.1 (CJMA-6) compared to the conductivity in NaCl solution of the same concentration. At the same time, our estimates show that the decrease in the membrane conductivity at this concentration should be between 2.1 and 1.3 times if we assume that the ratio between the counterion mobility (Cl^−^/H_2_PO_4_^−^ and Cl^−^/HPO_4_^2−^) is the same as in a solution. We hypothesize that a significant decrease in conductivity of the ASE membrane, when passing from a NaCl solution to a NaH_2_PO_4_ solution, is caused mainly by steric hindrances in the transport of large, highly hydrated phosphoric acid anions in these membranes. The sharp decrease in CJMA-6 membrane conductivity can be caused by the formation of the bound species between weakly basic amino groups and proton-containing phosphoric acid anions.

The neutral and negatively charged bound species formation, apparently, causes a change in the shape of current-voltage curves of the CJMA-6 membrane in Na_x_H_(3−x)_PO_4_ solutions compared to ASE and CJMA-3 membranes. In the case of the Na_x_H_(3−x)_PO_4_ with pH 10 ± 0.2, the shape of the CJMA-6 current–voltage curve becomes similar to the well-known curves for bipolar membranes. We explain this experimental fact by the formation of a bipolar junction in the CJMA-6 membrane at the boundary with a depleted solution. The negatively charged bound species and positively charged strongly basic fixed amino groups form this bipolar junction.

Specific interactions of weakly basic amino groups and phosphoric acid anions also explain the almost twofold increase in energy consumption for the electrodialysis recovery of phosphates from Na_x_H_(3−x)_PO_4_ solution with pH 4.4 ± 0.1 in the case of using the CJMA-6 membrane compared to CJMA-3.

Thus, it is already becoming clear that the use of anion-exchange membranes with weakly basic fixed amino groups is undesirable for the electrodialysis processing of phosphate-containing solutions.

## Figures and Tables

**Table 1:** Some characteristics of the studied membranes.

Membrane	Ion-Exchange Capacity of Wet Membrane, mmol/g_wet_	Water Content, gH_2_O/g_dry_, %	Thickness in 0.02 M NaCl Solution, µm	*f*_2_ in NaCl Solution	$\bar{Q},$ mmol/g_wet_
CJMA-3	0.57 ± 0.05 [48]	17 ± 1 [48]	151 ± 5	0.27 ± 0.02	0.8 ± 0.1
CJMA-6	0.90 ± 0.05 [48]	18 ± 1 [48]	120 ± 3	0.32 ± 0.02	1.3 ± 0.1
ASE	1.93 ± 0.05 [28]	20 ± 1	150 ± 5	0.06 ± 0.02	2.0 ± 0.1

**Table 2:** Some characteristics of the studied membranes.

Membranes	$\kappa^{*}$ in 0.04 M Solution, mS cm^−1^		$\kappa_{NaCl}^{*}/\kappa_{NaH_{2}PO_{4}}^{*}$
	NaCl	NaH_2_PO_4_	
CJMA-3	1.1 ± 0.05	0.6 ± 0.05	1.8 ± 0.1
CJMC-6	1.8 ± 0.05	0.4 ± 0.05	4.5 ± 0.1
ASE	3.8 ± 0.05	1.1 ± 0.05	3.3 ± 0.1

## Supplementary Materials

The following supporting information can be downloaded at: https://www.mdpi.com/article/10.3390/polym15102288/s1, Figure S1: Optical images of surfaces and cross-sections of CJMA-3 (a), CJMA-6 (b) and ASE (c) membranes; Figure S2: SEM images of (a) surfaces and (b) heterogeneous MK-40 membrane (d). The heterogeneous membrane MA-41 has a structure similar to that of MK-40; Figure S3: The distribution of species of the orthophosphoric acid (in mole fractions) vs. the pH of the solution; Figure S4: Schematic design of the experimental setup (a) and plexiglass frames with special comb-shaped guides that separate the membranes (b): a flow-through four-compartment electrodialysis cell containing an anion-exchange membrane under study (AEM*) and two auxiliary membranes, an anion-exchange and a cation-exchange membranes; tank with 0.02 M electrolyte solutions (1); additional tank (2) for determination of ion transport numbers; valves (3, 4); the Luggin capillaries (5); Ag/AgCl electrodes (6); platinum polarizing the working and counter electrodes (7); Autolab PGSTAT100N (8); flow-through cell with a pH combination electrode (9); pH meter pHM120 MeterLab (10) connected to computer; pH meter (10); combined electrode for pH measurements (11) connected to pH meter (10); conductivity cell (12) connected to a conductometer; titration device (13) for maintaining a constant pH in the solution circulating through tank (2); desalination compartment (14); the solid purple lines show schematic concentration profiles in two neighboring compartments separated by the membrane under study; Figure S5: Cross-section of the ion exchange membrane volume in the framework of microheterogeneous model; Figure S6: Concentration dependences of the conductivity of ASE, CJMA-3, and CJMA-6 membranes in NaCl solutions with pH 5.5 (a) and in NaH_2_PO_4_ solutions with pH 4.4 (b) presented in logarithmic coordinates. The multiplier in front of x corresponds to the value of f2; Table S1: The values of pKa (at 25 °C) of the orthophosphoric acid; Table S2: The characteristics of the studied electrolytes; Table S3: The found values of the Leveque limiting current densities for 0.02 M Na_x_H_(3−x)_PO_4_ solutions under study; References [1,4,7,9,10,13,14,15,16,17,18,19,20,21,23,24] are cited in the supplementary materials.
