# Supplementary material for: How Chemical Nature of Fixed Groups of Anion-Exchange Membranes Affects the Performance of Electrodialysis of Phosphate-Containing Solutions?
Source: Polymers (Basel). 2023 May 12;15(10):2288. doi: 10.3390/polym15102288 (PMC10221651; doi:10.3390/polym15102288)
Supplement: Supplementary file 1 [file polymers-15-02288-s001.zip › polymers-2337805-supplementary.pdf]

How Chemical Nature of Fixed Groups of Anion-Exchange Membranes affects the Performance of Electrodialysis of Phosphate-Containing Solutions?

Natalia Pismenskaya [1], Olesya Rybalkina [1], Ksenia Solonchenko [1], Evgeniia Pasechnaya [1], Veronika Sarapulova [1], Yaoming Wang [2], Chenxiao Jiang [2], Tongwen Xu [2], Victor Nikonenko [1*]

[1]Kuban State University, 149, Stavropolskaya Str., Krasnodar, 350040, Russian Federation; n_pismen@mail.ru (N.P.); olesia93rus@mail.ru (O.R.); sol.ksenia17@yandex.ru (K.S.); evgpasechnaya@yandex.ru (E. P.); vsarapulova@gmail.com (V. S.); v_nikonenko@mail.ru (V.N.)
[2]Department of Applied Chemistry, Anhui Provincial Engineering Laboratory of Functional Membrane
Science and Technology, School of Chemistry and Materials Science, University of Science and Technology of China, Hefei 230026, China; ymwong@ustc.edu.cn (W.Y.); jcx11@ustc.edu.cn (J.Ch.); twxu@ustc.edu.cn (X.T.).
* Correspondence: v_nikonenko@mail.ru; Tel.: +7-918-41-45-816

Supplementary materials

S1. Membranes

The commercial CJMA-3 [1] and experimental CJMA-6 homogeneous AEMs are manufactured by the Hefei Chemjoy Polymer Materials Co. Ltd. (Hefei, China). The generic name of this type of membrane CJMAED on the manufacturer's website emphasizes their possible use in electrodialysis. Their ion-exchange matrix contains polyvinylidene fluoride, PVDF (CJMA-3), or polyolefin (CJMA-6) functionalized with quaternary ammonium groups [2]. The matrix is cross-linked through the side chains [3]. These membranes are produced by the casting method and are reinforced with polyethylene terephthalate, PET (trademarks: Terylene, or Lavsan, or Dacron) cloth by hot rolling. The CJMAED membranes differ from each other in the type of polymer matrix, in the degree of cross-linking and in the concentration of fixed groups. These membranes are less costly compared with other anion-exchange membranes on the world market.

The manufacturer of the homogeneous Neosepta ASE anion exchange membrane [4], does not provide information on the chemical structure of this membrane. At the same time Chen et al. [5] reported that this membrane has a base polymer of styrene (crosslinked with divinyl-benzene) with strongly basic groups as the positive fixed charges; the backing fabric is fabricated from the mixture of polyethylene and polypropylene. (Fig. S1c).

Heterogeneous cation exchange MK-40 membrane and anion exchange MA-41 membrane are made by hot rolling of milled cation-exchange resins KU-2-8 and anion – exchange resin AV-17-8, as well as a high-density polyethylene powder. Then reinforcing nylon mesh is introduced (Fig. 2a) using the hot pressing method [6]. The size of ion-exchange resin particles ranges from 5 to 50 μm. The adhesion between individual resin particles, polyethylene,

and the reinforcing cloth is low, resulting in gaps that form macropores (about 1 μm in size) when the membrane contacts solutions [7]. The resin particles are evenly distributed throughout the IEM (Fig. 2b); their tops extend beyond the polyethylene-coated surface of heterogeneous membranes (Fig. 2b). The resin is a copolymer of styrene and divinylbenzene (8%). It contains sulphonate fixed groups (KU-2-8) or quaternary ammonium bases as fixed functional groups and small amounts of weakly basic primary and secondary amines (AV-17-8).

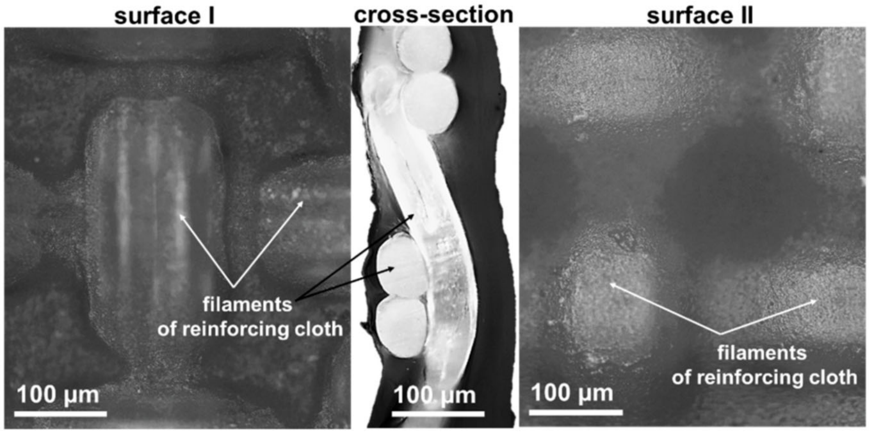

(a)

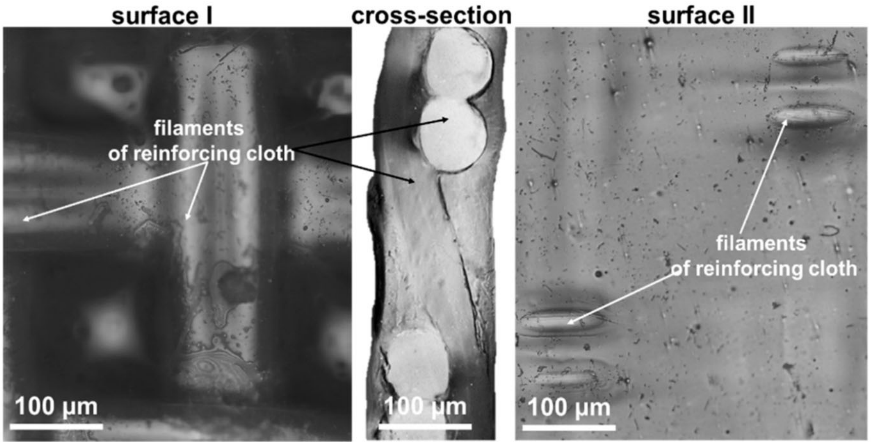

(b)

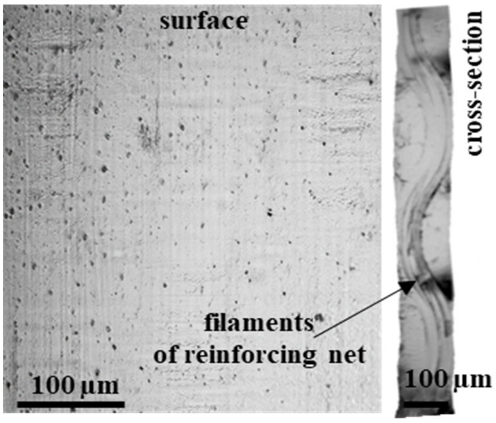

(c)

Figure S1. Optical images of surfaces and cross-sections of CJMA-3 (a), CJMA-6 (b) and ASE(c) membranes.

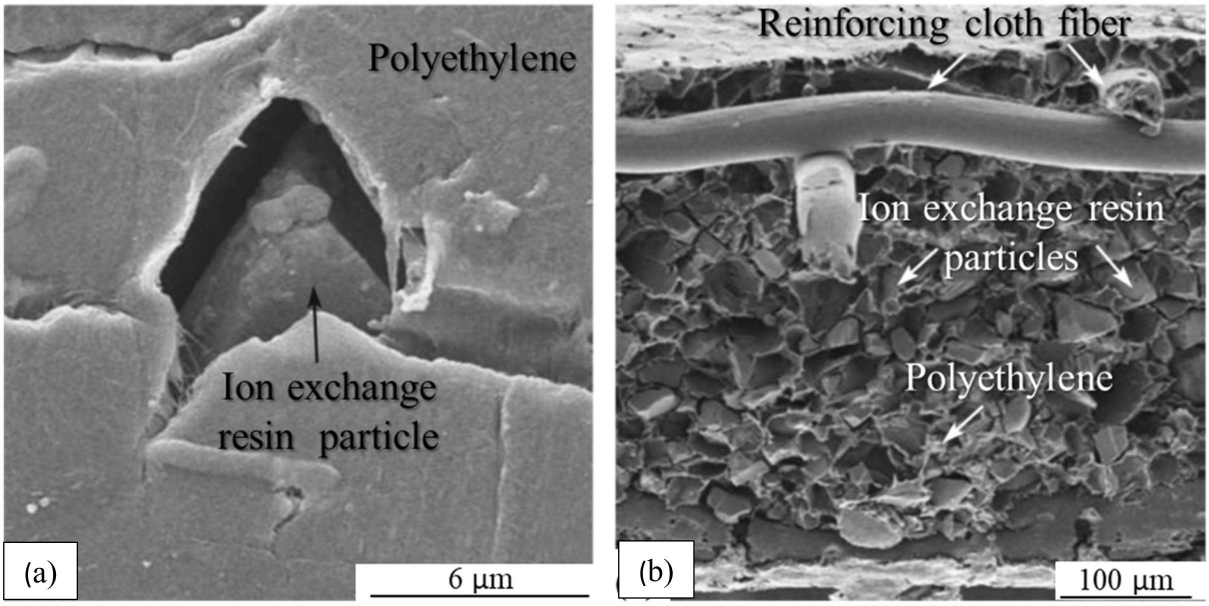

Figure S2. SEM images of (a) surfaces and (b) heterogeneous MK-40 membrane (d). The heterogeneous membrane MA-41 has a structure similar to that of MK-40.

S2 Solutions

$Na_xH_{(x-3)}PO_4$ are the salts of the tribasic orthophosphoric acid. The latter has the following structure

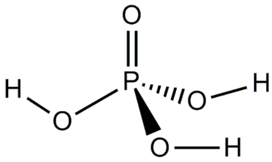

The proton-transfer reactions between water molecules and orthophosphoric acid species (the case of orthophosphoric acid with general formula H_nA, where the maximum value of n is 3) are presented as follows:

$$\mathrm{H_2A^- + H_2O \underset{k_{-1}}{\overset{k_1}{\rightleftarrows}} H_3A + OH^-}; \qquad K_{b1} = \frac{c_{H_3A}c_{OH^-}}{c_{H_2A^-}} \tag{S1}$$

$$\mathrm{H_3A + H_2O \underset{k_{-2}}{\overset{k_2}{\rightleftarrows}} H_2A^- + H_3O^+}; \qquad K_{a1} = \frac{c_{H_2A^-}c_{H^+}}{c_{H_3A}} \tag{S2}$$

$$\mathrm{HA^{2-} + H_2O \underset{k_{-3}}{\overset{k_3}{\rightleftarrows}} H_2A^- + OH^-}; \qquad K_{b2} = \frac{c_{H_2A^-}c_{OH^-}}{c_{HA^{2-}}} \tag{S3}$$

$$\mathrm{H_2A^- + H_2O \underset{k_{-4}}{\overset{k_4}{\rightleftarrows}} HA^{2-} + H_3O^+}; \qquad K_{a2} = \frac{c_{HA^{2-}}c_{H^+}}{c_{H_2A^-}} \tag{S4}$$

$$\mathrm{A^{3-} + H_2O \underset{k_{-5}}{\overset{k_5}{\rightleftarrows}} HA^{2-} + OH^-}; \qquad K_{b3} = \frac{c_{HA^{2-}}c_{OH^-}}{c_{A^{3-}}} \tag{S5}$$

$$\mathrm{HA}^{2-} + \mathrm{H_2O} \underset{k_{-6}}{\overset{k_6}{\rightleftarrows}} \mathrm{A}^{3-} + \mathrm{H_3O}^{+}\,; \qquad K_{a3} = \frac{c_{A^{3-}} c_{H^+}}{c_{HA^{2-}}} \tag{S6}$$

Fig. S3. shows the distribution of species of the polybasic acids under study (in mole fractions) vs. the pH of the solution. This distribution is calculated using the appropriate equilibrium equations and the pK_a values presented in Table S1.

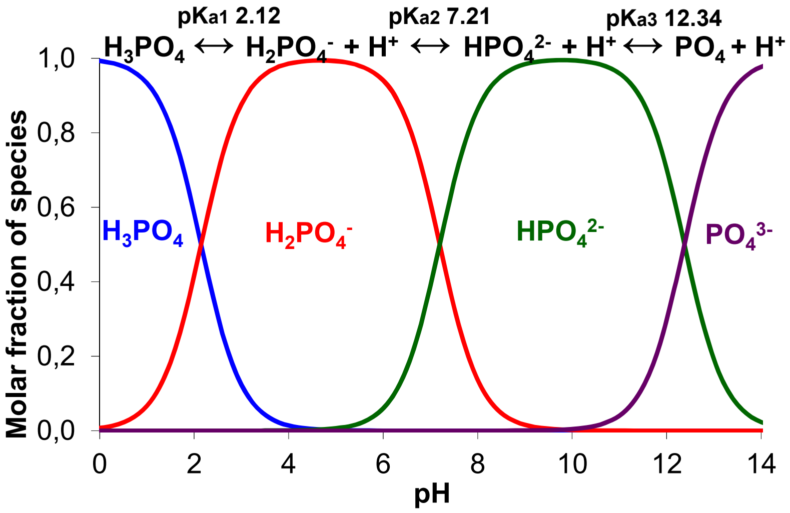

Figure S3. The distribution of species of the orthophosphoric acid (in mole fractions) vs. the pH of the solution.

Calculation of the rate constants

There are relationships between the pseudo-unimolecular forward rate constants and backward rate constants, which involve the equilibrium constants, the acid dissociation (K_a) or the base ionization (K_b) constants. For example, in the case of reactions (S1) and (S2), we have[8,9]:

$$\frac{k_1'}{k_{-1}} = K_{b1} = \frac{K_w}{K_{a1}}\,; \qquad \frac{k_2'}{k_{-2}} = K_{a1} = \frac{K_w}{K_{b1}} \tag{S7}$$

where K_w (equal to 10^{-14} mol^2 dm^{-6}) is the water dissociation constant; K_{ai} (mol dm^{-3}) is defined for each step of dissociation i=1, 2, 3 by Eqs. (S2), (S4) and (S6), respectively; $K_{bi} = K_w / K_{ai}$. Equations, similar to Eqs. (S7), can also be written for the 2-nd and 3-rd dissociation steps. The pseudo-unimolecular forward rate constants k_1' and k_2' are obtained, when the concentration of water, c_{H_2O}, is considered as a constant whose value is taken into account in the value of these constants: $k_1' = k_1 c_{H_2O}$, $k_2' = k_2 c_{H_2O}$ [8,10].

The values of the dissociation constants, K_{ai}, used in the calculations are given in Table S1. The values of the backward rate constants k_{-1} and k_{-2} are both taken equal to 10^{10} dm^{-3} mol^{-1} s^{-1}[8].

Table S1

The values of pK_a (at 25 °C) of the orthophosphoric acid [11] various species, which may be present in the membrane systems under study.

Substance	pK_{a1}	pK_{a2}	pK_{a3}
H_3PO_4	2.12	7.21	12.34

S3. Methods

The thickness of the swollen membrane (d, μm) was controlled by a high-precision digital micrometer MKC-25 0.001 with an accuracy of 1 μm. The membrane thickness was obtained by averaging the results of 10 measurements made at various points of the sample under study.

The total exchange capacity (Q) of the anion exchange membranes (AEM) under study was found as follows. The AEM samples were placed in a 0.1 M HCl solution. The samples were abundantly washed with water; they were immersed in a 1.0 M HNO_3 solution during 12 h at 25°C. The ion-exchange capacity was determined by the chloride content in the equilibrium solution using the following equation:

$$Q = \frac{C_{AgNO_3} V_{AgNO_3}}{m_{wet}} \quad (S8)$$

where C_{AgNO_3} is the concentration of $AgNO_3$; V_{AgNO_3} is the volume of $AgNO_3$ consumed for the titration of Cl^- ions exchanges with the NO_3^- ions, and m_{wet} is the weight of swollen membrane.

Water content (W, %) of the membranes was determined by the gravimetric method. Before the experiment, the samples were equilibrated with a 0.02 eq L^{-1} NaCl solution at 25 ± 1 °C for 24 h. After equilibration, samples were taken out from the solution and the film of liquid was removed from the samples' ends and surfaces using filter paper.

Weights of wet, m_{wet}, and dry, m_{dry}, samples were obtained using an MB25 Ohaus moisture analyzer. The evaporation of water was carried out at a temperature of 50 °C to a constant weight of the sample.

The water content W, % was calculated by the formula:

$$W = \frac{m_{wet} - m_{dry}}{m_{dry}} \times 100\% \quad (S9)$$

Electrical conductivity of AEMs (κ*) was determined by a differential method using a clip cell [12] and a AKIP 6104 immitance meter (Motech Industries Inc., Taiwan) at an AC frequency of 1 kHz. All samples were studied in 0.02–1.0 eq L^{-1} solutions of NaCl and NaH_2PO_4 (pH 4.4±0.1) starting from the lowest concentration.

The conductivity of the membranes (κ*) was found by the formula:

$$\kappa^* = \frac{d_m}{R_{m+s} - R_s} \quad (S10)$$

where R_{m+s} is the resistance of the membrane in solution and R_s is the resistance of the solution alone.

The current-voltage characteristics (CVC) and the pH difference between the outlet and inlet solutions of the desalination compartment. CVCs were recorded at the current sweep of 0.02 mA s^{-1}. The working area of measuring Ag/AgCl electrodes immersed in the solution identical to the feed solution is 11.2 cm^2. In the case of phosphate, tartrate or citrate containing solution, a small amount of NaCl (~1%) was added to the solution to ensure the stable operation of the measuring electrodes. The initial volume (before the experiment) of the feed solution in tank (1) and the hoses was 5 L. The feed solution was pumped from tank (1) through all the cell compartments; then, it returned into the same tank. The salt concentration in the feed solution circulating through tank (1) changed very little (<1%) during one experimental run due to the relatively large volume of this solution.

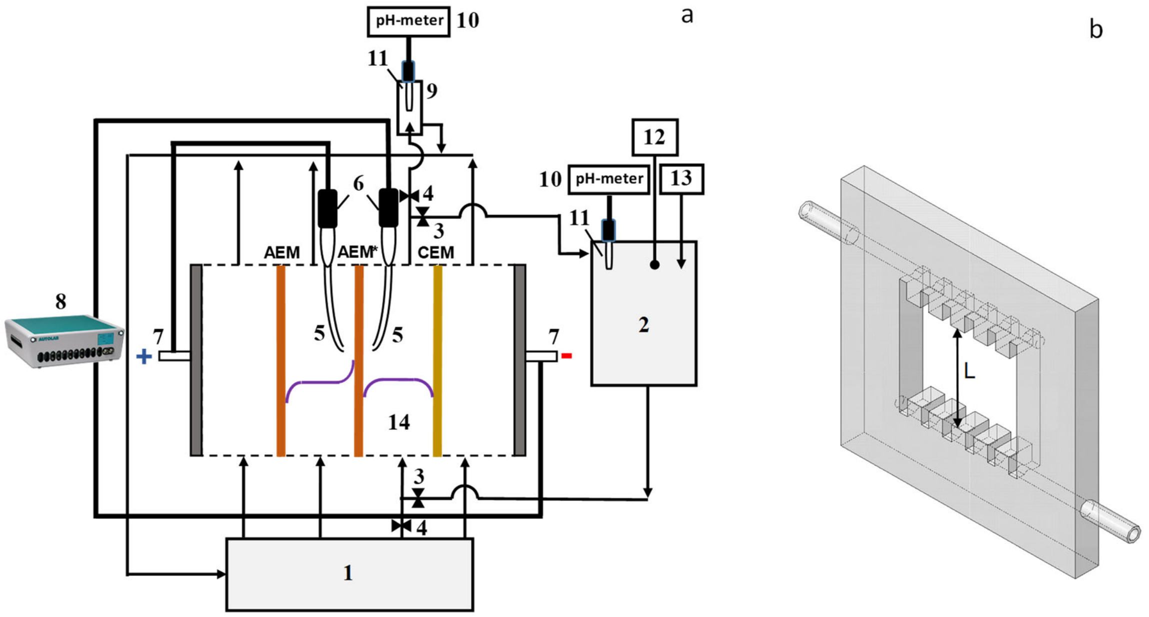

Figure S4. Schematic design of the experimental setup (a) and plexiglass frames with special comb-shaped guides that separate the membranes (b): a flow-through four-compartment electrodialysis cell containing an anion-exchange membrane under study (AEM*) and two auxiliary membranes, an anion-exchange and a cation-exchange membranes; tank with 0.02 M electrolyte solutions (1); additional tank (2) for determination of ion transport numbers; valves (3, 4); the Luggin capillaries (5); Ag/AgCl electrodes (6); platinum polarizing the working and counter electrodes (7); Autolab PGSTAT100N (8); flow-through cell with a pH combination electrode (9); pH meter pHM120 MeterLab (10) connected to computer; pH meter (10); combined electrode for pH measurements (11) connected to pH meter (10); conductivity cell (12) connected to a conductometer; titration device (13) for maintaining a constant pH in the solution circulating through tank (2); desalination compartment (14); the solid purple lines show schematic concentration profiles in two neighboring compartments separated by the membrane under study.

The potential drop over the membrane under study (AEM*) measured using Luggin's capillaries (5) is a function of the distance between the capillary tip and the membrane as well as

the ohmic resistance produced by the membrane [13]. When the cell is disassembled for replacing the membrane, these parameters change. To exclude this ambiguity, instead of the total potential drop, $\Delta\varphi$, we use the corrected potential drop, $\Delta\varphi'$, defined as follows [14]:

$$\Delta\varphi' = \Delta\varphi - iR_{ef} \tag{S11}$$

where R_{ef} (Ohm cm^2) is the effective resistance, which is found by extrapolation $i \to 0$ in the coordinates $i - d\varphi/di$, using the initial part of CVC [12].

Electrodialysis processing of the phosphate solution was carried out using the cell presented in Fig. S4. The difference with the measurements of CVC is in the fact that desalination compartment (14) is fed from additional tank (2). The volume of the solution circulating through the desalination compartment and tank (2) is 0.1 L, which is essentially less than the volume of the solution circulating through tank (1), the concentration and electrode compartments. During one experimental run, in conditions where the current density in the electrodialysis cell potential is kept constant, the salt concentration in the diluate stream decreased with time. Since the rates of generation of H^+ and OH^- ions at the CEM and AEM forming the desalination compartment are different, pH of the feed solution changed with time. Namely, it became acidic in the studied cases. In order to keep a constant pH value of the feed solution, a 0.1M solution of NaOH was added into tank (2) through microcapillary (13). The rate of decrease in the salt concentration of the solution in tank (2), *dC/dt*, is found by using the measured values of conductivity, κ, of this solution (using submersible conductometric cell (12)) and taking into account the known constant pH value. The potential drop was measured during the entire experiment with an interval of 300 s. The measurements were carried out using Luggin capillaries (5), located near the center of the polarizable surfaces of the membrane under study. The distance between the membrane surface and the tip of the capillary was about 0.8 mm.

The desalination degree, $\boldsymbol{\gamma_D}$, and the component *i* recovery degree, $\boldsymbol{\gamma_i}$, were found by the equations:

$$\gamma_D = \frac{\kappa^0 - \kappa^t}{\kappa^0} \tag{S12}$$

$$\gamma_i = \frac{c_i^0 - c_i^t}{c_i^0} \tag{S13}$$

where the indices «0» and «t» correspond to the characteristics of the diluate stream at the beginning and after a given time of electrodialysis.

Electric charge, *Q*, and energy consumption, *W*, spent on the desalination of the solution was determined as:

$$Q = I_{av}t \quad (S14)$$

$$W = \int I(t)\Delta\varphi(t)dt \quad (S15)$$

where I_{av} – is the average current intensity over the period. The value of W was calculated using equation (S15), according to the dependences of $I(t)$ and $\Delta\varphi(t)$ measured using the Autolab PGSTAT100 electrochemical complex. Integration in accordance with equation (S15) was carried out from the beginning of the ED process, t=0, to the time $t=t'$, when the desired desalination degree was reached (50%).

The current efficiency were calculated using the equation:

$$\eta = \frac{z_i F \overline{V}(C_{0i} - C_{ti})}{n\int_0^t I_{tot}(t)\,dt} \quad (S16)$$

S4 Microheterogeneous model

Theory

According to the microheterogeneous model, an ion exchange membrane (IEM) is considered as a two-phase system involving a gel phase and intergel spaces filed with an equilibrium electroneutral solution. The volume fractions of these phases are f_1 and f_2, ($f_1 + f_2 = 1$).

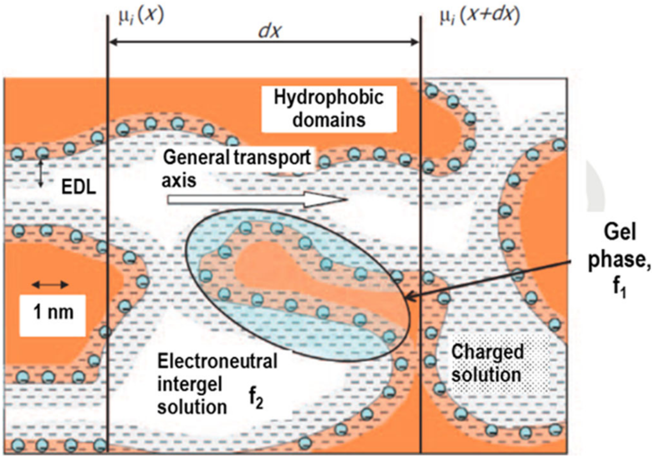

Figure S5. Cross-section of the ion exchange membrane volume in the framework of microheterogeneous model [15].

The gel phase is a microporous swollen medium (Figure S5). It includes the polymer matrix, which bears charged fixed groups, and the charged solution of mobile counterions and, in a smaller number, coions that compensate for the charge of fixed groups. The reinforcing cloth fibers and the inert filler (polyethylene) are also included in the gel phase. The second phase

includes the solution in the central part of the meso- and macropores and in the structural defects of the membrane.

In the first approximation (when the presence of coions in the gel phase is neglected), the electrical conductivity of this phase is considered to be constant, depending on the counterion diffusion coefficient in the gel phase of the membrane, $\overline{D_i}$, and on its exchange capacity, $\bar{Q}$:

$$\bar{\kappa} = \frac{z_i \bar{D}_i \bar{Q} F^2}{RT}, \tag{S17}$$

where F is the Faraday constant, R is the universal gas constant, T is the temperature, and z_i is the counterion charge. The value is related to the ion-exchange capacity $\bar{Q}$ of the membrane by $\bar{Q} = Q/f_1$. The numerical value of $\bar{\kappa}$ can be determined from the value of the membrane electrical conductivity (κ^*) at the isoconductivity point in which the conductivities of the membrane (κ^*) and the solution (κ) are identical. It is clear that according to Equation (S17), the following equality holds in this case: κ^*= κ =$\bar{\kappa}$. For commercial membranes and strong electrolytes, the concentration at this point (C_{iso},) is not far from 0.05 mol L^{-1} solution: at $C > C_{iso}$, the conductivity of the membrane is higher than that of the solution, $\kappa^* > \kappa$, and at $C > C_{iso}$, $\kappa^* < \kappa$.

According to the microheterogeneous model [15], within the concentration range $0.1c_{iso} < c < 10c_{iso}$, and under condition that parameter α is not too great ($|\alpha| \leq 0.2$), the electrical conductivity of an IEM, κ^*, can be expressed as:

$$\kappa^* = \bar{\kappa}^{f_1} \kappa^{f_2} \tag{S18}$$

where the conductivity of the "intergel" solution, κ, is assumed to be equal to that of the external equilibrium solution.

According to Equation (S18), the $\lg(\kappa^*)$ vs $\lg(\kappa)$ dependence gives a straight line, which is well confirmed by experimental data. The slope of the line is equal to f_2 (if $\bar{\kappa}$ is assumed constant). The term b of the equation y=ax+b approximating the trend line of the dependence $\lg(\kappa^*)$ vs $\lg(\kappa)$is equal to $f_1 \lg\bar{\kappa}$.

In the general case, the conductivity and other transport parameters are obtained after expressing the effective membrane conductance coefficient, L_i^*, through the conductance coefficients of the individual constituent phases in the following form:

$$L_i^* = \left[f_1 \left(L_i^g\right)^\alpha + f_2 (L_i^s)^\alpha\right]^{1/\alpha}, \tag{S19}$$

where L_i^g refers to the gel phase, and L_i^s to the inter-gel electroneutral solution; α is the structural parameter depending on the position of the phases with respect to the axis of transport: when the phases are parallel to this axis, α = 1; when they are in serial disposi-tion, α = −1; in other cases −1 < α < 1.

When describing the transport in the gel phase, we apply the assumptions of the Teorell-Meyer-Sievers (TMS) model. The properties of the intergel solution are assumed the same as those of the bathing equilibrium solution; the Nernst-Planck and the local electroneutrality assumption are used there. L_i^s and L_i^g are expressed as functions of the ionic diffusion coefficients, D_i^s and D_i^g, and the concentrations, c_i^s and c_i^g in the corresponding phase:

$$L_i^s = D_i^s c_i^s / RT, \qquad L_i^g = D_i^g c_i^g / RT \tag{S20}$$

Concentrations c_i^s and c_i^g are linked by the Donnan relation:

$$\frac{(\bar{c}_1)^{1/z_1}}{(\bar{c}_A)^{1/z_A}} = K_D \frac{(c_1)^{1/z_1}}{(c_A)^{1/z_A}}, \tag{S21}$$

where K_D is the Donnan equilibrium coefficient; $\bar{c}_i$ and c_i are the concentrations of ion i in the gel phase and the intergel solution, respectively; subscript "*1*" is used for the counterion; "*A*", for the coion; c_A refers to the coion concentration in the inter-gel solution.

Usually the concentration of fixed ions (IEC, Q) in ion-exchange materials is rather high, close to 1 mol (L wet membrane)$^{-1}$ or higher. Co-ions are therefore strongly excluded, so that their concentration in the gel phase is small compared to $\bar{Q}$, at least in relatively dilute solutions. When assuming $\bar{c}_- \ll \bar{Q}$ and $\bar{c}_+ \approx \bar{Q}$, Equation (S21) can be simplified. In the case of a symmetrical electrolyte ($z_+ = -z_- = z$) one gets:

$$\bar{c}_A = \frac{K_D^z}{\bar{Q}} c_A^2, \qquad \bar{c}_1 = \bar{Q} + \bar{c}_A \tag{S22}$$

The ions are present in both phases; the partition coefficient, K_s, involving the co-ion concentration in the membrane, c_A^* , can be found using Equation (S22):

$$K_s = \frac{c_A^*}{c_A} = f_1 \frac{K_D}{Q^g} c_A + f_2, \tag{S23}$$

in which the first term on the right-hand side stands for the contribution of the gel phase, and the second term, for that of the inter-gel solution. Q^g and K_D are the IEC of the gel phase and the Donnan coefficient, respectively. Despite of small value of f_2 (typically less than 0.1 in homogeneous membranes, and close to 0.2 in heterogeneous ones), the sorption of electrolyte by the inter-gel spaces is dominant, especially in diluted solutions, because of the co-ion exclusion from the gel involving EDL in micro- and mesopores is small. For conventional IEMs, Equation (S22) is verified for external concentrations up to 1–2 eq L^{-1}.

After finding the values of L_i^*, the membrane conductivity, diffusion permeability and ion transport numbers in the membrane can be expressed through L_i^* as follows:

$$\kappa^* = (z_+^2 L_+^* + z_-^2 L_-^*) F^2 \tag{S24}$$

$$t_i^* = \frac{z_i^2 L_i^*}{z_+^2 L_+^* + z_-^2 L_-^*} = \frac{z_i^2 L_i^* F^2}{\kappa^*}, i = +, - \tag{S25}$$

$$P^* = \frac{(z_+ L_+^* t_-^* + |z_-| L_-^* t_+^*)RT}{C}, \tag{S26}$$

where $Q = |z_i| c_i$ is the electrolyte concentration in the intergel solution expressed in eq L^{-1}.

Experimental data processing

The results of processing the concentration dependences of the conductivity of the studied membranes using a microheterogeneous model are shown in the figure S6.

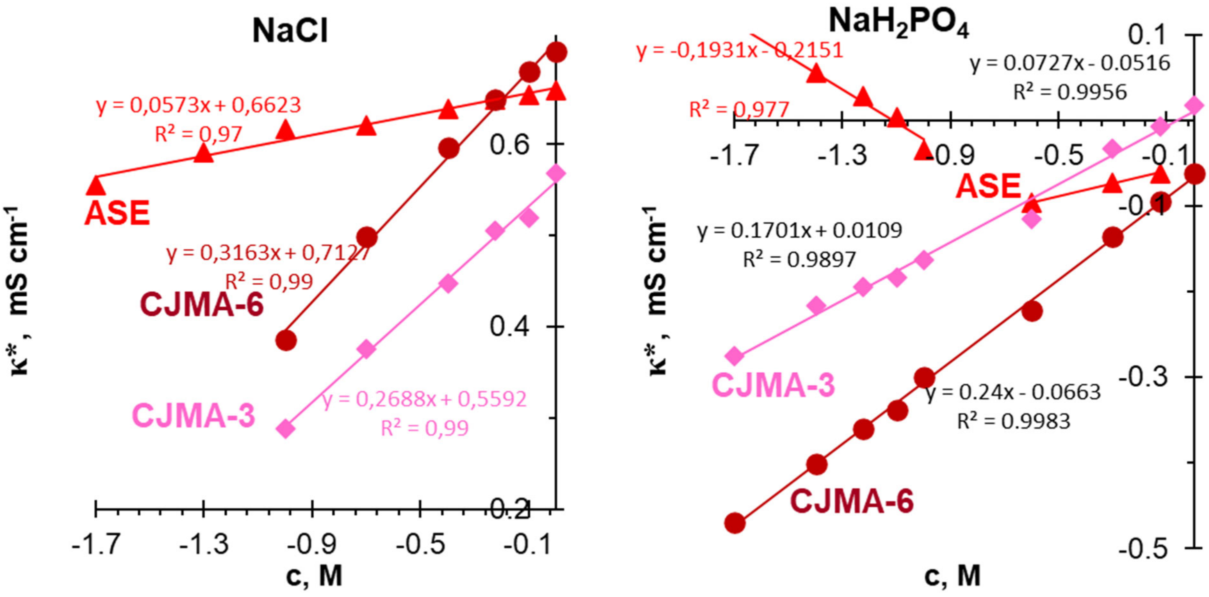

a b

Figure S6. Concentration dependences of the conductivity of ASE, CJMA-3, and CJMA-6 membranes in NaCl solutions with pH 5.5 (a) and in NaH_2PO_4 solutions with pH 4.4 (b) presented in logarithmic coordinates. The multiplier in front of x corresponds to the value of f_2.

S5. Theoretical limiting current calculations

The Lévêque limiting current density in NaCl solution

The Leveque equation [(S27) and equation (S28)] are used to estimate the theoretical limiting current, i_{lim}^{Lev}, and the thickness of the depleted diffusion layer, δ^{Lev} in NaCl solution:

$$i_{\lim}^{Lev} = \frac{z_1 F D c_1^0}{h(T_1 - t_1)} \left[1.47 \left(\frac{h^2 V_0}{LD} \right)^{1/3} \right] \tag{S27}$$

$$\delta^{Lev} = 0.71 h \left(\frac{LD}{h^2 V_0} \right)^{1/3} \tag{S28}$$

Here z_1 is the charge number of counterion 1, F is Faraday constant, D и t_1 are the diffusion coefficient of the electrolyte and the electromigration transfer number of the counterion at infinite dilution of the solution. The transport number of counterions in the membrane, T_1, was considered equal to one; c_1 is its molar concentration in the feed solution entering the DC, V_0 is the average linear velocity of the solution flowing between the membranes forming the DC, h is

the distance between the membranes, L is the length of the channel. These equations were obtained for the diffusion-convection heat transfer [16] and after were adapted to diffusion-convection mass transfer in electrode [17] and ion exchange membrane [18] systems. For 1: 1 electrolyte and laminar hydrodynamic regime. Note that the value 0.71 for the factor in the right-hand part of Eq. (S39) is given by the numerical solution of the 2D convection-diffusion problem whose asymptotic solution is expressed by Eq. (S38). However, often [19,20] Eq. (S28) is used with a factor 0.68, which is obtained using the Peers equation [21] and the approximation of Eq. (S27), where the second term in the brackets is neglected. It is worth noting that this equation is applicable only for relatively short channel lengths ($L \leq 0.02h^2V_0 / D$) [22].

The Lévêque limiting current density in a mixed electrolyte solution ($Na_xH_{(3-x)}PO_4$)

Let us consider a ternary electrolyte composed of two kinds of counterions, 1 and 2, and one kind of coion, a later on, we consider a solution of this ternary electrolyte in a diffusion layer adjacent to an ion-exchange membrane.

The Nernst-Planck equations for these ions read:

$$j_1 = -D_1\left(\frac{dc_1}{dx} + z_1c_1\frac{F}{RT}\frac{d\varphi}{dx}\right) \tag{S29}$$

$$j_2 = -D_2\left(\frac{dc_2}{dx} + z_2c_2\frac{F}{RT}\frac{d\varphi}{dx}\right) \tag{S30}$$

$$j_A = -D_A\left(\frac{dc_A}{dx} + z_Ac_A\frac{F}{RT}\frac{d\varphi}{dx}\right) \tag{S31}$$

After dividing each of the equations (S29)-(S31) by D_i, summing the results, and taking into account the electroneutrality condition

$$z_1c_1 + z_2c_2 + z_Ac_A = 0 \tag{S32}$$

we find:

$$\frac{j_1}{D_1} + \frac{j_2}{D_2} + \frac{j_A}{D_A} = -\frac{d\left(c_1 + c_2 + c_3\right)}{dx} = -\frac{\left(1 + \left|z_1 / z_A\right|\right)dc_1}{dx} - \frac{\left(1 + \left|z_2 / z_A\right|\right)dc_2}{dx} \tag{S33}$$

The last equality in Eq. (S33) is obtained after eliminating c_A using Eq. (S32). Since j_i do not change along the coordinate x in a stationary state, Eq. (S33) can be easily integrated over the thickness of the diffusion layer. If the current density is equal to the limiting one, the concentrations of all ions at the membrane surface are very close to zero. In this case, we can write:

$$\frac{j_{1\lim}^{theor}}{D_1}+\frac{j_{2\lim}^{theor}}{D_2}=\frac{\left(1+\left|z_1/z_A\right|\right)c_1^0}{\delta}+\frac{\left(1+\left|z_2/z_A\right|\right)c_2^0}{\delta}-\frac{j_{A\lim}^{theor}}{D_A} \tag{S34}$$

It follows from Eq. (S27) that the limiting flux density of counterion i can be represented as [23]:

$$j_{i\lim}^{theor}=\frac{\left(1+\left|z_i/z_A\right|\right)D_i c_i^0}{\delta}-\frac{\left|z_i\right|D_i c_i^0}{\left|z_A\right|D_A c_A^0}\cdot j_{A\lim}^{theor} \tag{S35}$$

The first term in Eq. (S35) shows what value the limiting flux density of counterion i would have if the membrane were impermeable to coions. The second term reflects the additional value of the counterion flux caused by the transfer of coions: when the coions appear in the depleted layer, they create an additional electric field that attracts counterions from the solution. This effect is called exaltation in the literature [16,24].

From Eq. (S36), it is easy to obtain an expression for the limiting current density:

$$i_{\lim}=z_1 j_1+z_2 j_2+z_A j_A=i_{\lim}^0+\left(\frac{T_{A_{\lim}}}{t_A}\right)i_{\lim} \tag{S36}$$

where $t_A=\frac{D_A z_A^2 c_A^0}{\sum_{i=1,2,A} D_i z_i^2 c_i^0}$ is the coion transport number in the bulk solution, $T_{A_{\lim}}=\frac{z_A j_{A\lim}^{theor}F}{i_{\lim}}$ is the coion effective transport number in the membrane at $i=i_{\lim}$,

$$i_{\lim}^0=\frac{\left[\left(1+\left|z_1/z_A\right|\right)D_1 z_1 c_1^0+\left(1+\left|z_2/z_A\right|\right)D_2 z_2 c_2^0\right]F}{\delta} \tag{S37}$$

is the limiting current density in the case of a membrane impermeable to coions. The term $\left(T_{A_{\lim}}/t_A\right)i_{\lim}$ in Eq. (S37) can be interpreted as the sum of the current carried by the coions $\left(T_A\cdot i_{\lim}\right)$, and the exaltation current of counterions. From Eq. (S37), we obtain:

$$i_{\lim}=\frac{i_{\lim}^0\cdot t_A}{t_A-T_A} \tag{S38}$$

The resulting Eq. (S37) generalizes the well-known Peers equation for a single electrolyte. Indeed, setting $c_2^0=0$ gives:

$$i_{\lim}=\frac{D\cdot z_1\cdot c_1^0\cdot F}{\left(T_1-t_1\right)\delta} \tag{S39}$$

where:

$$D=\frac{D_1 D_A\left(z_1+\left|z_A\right|\right)}{D_1 z_1+D_A\left|z_A\right|} \tag{S40}$$

is the electrolyte diffusion coefficient.

For a mixture of two single electrolytes with a common coion (a ternary electrolyte), one can obtain equation (S41), which is similar to equation (S39) [25]:

$$i_{\lim} = \frac{D_{ter} \cdot |z_A| \cdot c_A^0 \cdot F}{\left(t_A - T_A\right)\delta} \tag{S41}$$

with D_{ter}, the effective diffusion coefficient of ternary electrolyte,

$$D_{ter} = \left[\left(1+\left|\frac{z_1}{z_A}\right|\right)D_1N_1 + \left(1+\left|\frac{z_2}{z_A}\right|\right)D_2N_2\right]\cdot t_A = \frac{\left[\left(z_1+|z_A|\right)D_1z_1c_1^0 + \left(z_2+|z_A|\right)D_2z_2c_2^0\right]D_A}{\sum\limits_{i=1,2,A} D_i z_i^2 c_i^0} \tag{S42}$$

$N_i = \dfrac{z_i c_i^0}{z_A c_A^0}$ is the equivalent fraction of counterion i in the bulk solution. It is easy to see that in the case, where the concentration of counterion 2 is zero, $c_2^0 = 0$, Eq. (S42) is reduced to Eq. (S41).

For a single electrolyte, the Leveque equation (Eq. (27)), allows calculating the limiting current density and diffusion layer thickness as functions of the (single) electrolyte diffusion coefficient, solution flow rate, distance between the membranes and membrane length. It can be assumed that this equation remains valid in the case of ternary electrolyte (e.g. *Na_2HPO_4 + NaH_2PO_4*) if the value determined by Eq. (S42) is used as the electrolyte diffusion coefficient.

$$\delta^{Lev} = 0.68h\left(\frac{LD}{h^2V_0}\right)^{1/3} \tag{S43}$$

Table S2 summarizes some of the characteristics of the studied electrolytes, which are used to calculate the limiting currents.

Table S2

Some of the characteristics of the studied electrolytes, which are used to calculate the limiting currents.

Electrolyte	Diffusion coefficients at infinite dilution, $D_i \times 10^5$, cm^2 s^{-1}				Transport numbers at infinite dilution, t_i		
	cation	anion		electrolyte	cation	anion	
		singly charged	doubly charged			singly charged	doubly charged
NaCl	1.334 [26]	2.032 [26]		1.61	0.396	0.604	
NaH_2PO_4		0.959 [26]		1.12	0.581	0.419	
Na_2HPO_4		-	0.759 [26]				0.456

Calculations made using equation S22 give the following value D_{ter}: $1.12 \cdot 10^{-9}$ cm^2 s^{-1} (pH 4.4 ± 0.1), $1.20 \cdot 10^{-9}$ cm^2 s^{-1} (pH 6.6 ± 0.1) and $1.38 \cdot 10^{-9}$ cm^2 s^{-1} (pH 10.0 ± 0.2). It is calculated under the assumption that Na^+ is the only coion.

The found values of the Leveque limiting current densities are presented in the table S3.

Table S3

The found values of the Leveque limiting current densities for 0.02 M $Na_xH_{(3-x)}PO_4$ solutions under study

pH	Molar concentration, M				i_{lim}^{Lev}, mA cm^{-2}
	NaH_2PO_4	Na_2HPO_4	Na_3PO_4	NaCl	
4.4 ± 0.1	1.99 10^{-2}	3.16 10^{-5}	3.35 10^{-13}	-	1.64
6.6 ± 0.1	1.60 10^{-2}	4.03 10^{-3}	6.77 10^{-9}	-	2.04
10.0 ± 0.2	1.98 10^{-5}	1.98 10^{-2}	1.33 10^{-4}	-	3.58
5.6 ± 0.1	-	-	-	2.0 10^{-2}	3.07

References

1. Hefei ChemJoy Polymer Materials Co Available online: www.cj-membrane.com/display/369191.html.
2. Wang, Y.; Zhang, Z.; Jiang, C.; Xu, T. Recovery of gamma-aminobutyric acid (GABA) from reaction mixtures containing salt by electrodialysis. *Sep. Purif. Technol.* **2016**, *170*, 353–359, doi:10.1016/j.seppur.2016.07.002.
3. Yan, H.; Wang, Y.; Xu, T. Developing Ion Exchange Membrane for Treating High Salinity Water Using Electrodialysis, Tianjin, China, 2019.
4. Astom Detailed Specification of IEMs Produced Astom Corporation Available online: http://www.astom-corp.jp/en/product/10.html.
5. Chen, G.Q.; Wei, K.; Hassanvand, A.; Freeman, B.D.; Kentish, S.E. Single and binary ion sorption equilibria of monovalent and divalent ions in commercial ion exchange membranes. *Water Res.* **2020**, *175*, 115681, doi:10.1016/j.watres.2020.115681.
6. Nefedova, G.Z.; Klimova, Z.G.; Sapoznikova, G.S. Ion-Exchange Membranes, Granulates, Powders. *Catalogue* **1977**.
7. Berezina, N.P.; Kononenko, N.A.; Dyomina, O.A.; Gnusin, N.P. Characterization of ion-exchange membrane materials: Properties vs structure. *Adv. Colloid Interface Sci.* **2008**, *139*, 3–28, doi:10.1016/j.cis.2008.01.002.
8. Simons, R. Electric field effects on proton transfer between ionizable groups and water in

ion exchange membranes. *Electrochim. Acta* **1984**, *29*, 151–158, doi:10.1016/0013-4686(84)87040-1.

9. Simons, R. Water splitting in ion exchange membranes. *Electrochim. Acta* **1985**, *30*, 275–282, doi:10.1016/0013-4686(85)80184-5.
10. Zabolotskii, V.I.; Shel'deshov, N. V; Gnusin, N.P. Dissociation of Water Molecules in Systems with Ion-exchange Membranes. *Russ. Chem. Rev.* **1988**, *57*, 801–808, doi:10.1070/RC1988v057n08ABEH003389.
11. Lide, D.R.; Baysinger, G.; Berger, L.I.; Kehiaian, H. V; Roth, D.L.; Zwillinger, D.; Goldberg, R.N.; Haynes, W.M. *CRC Handbook of Chemistry and Physics*; CRC Press: New York, 1997;
12. Lteif, R.; Dammak, L.; Larchet, C.; Auclair, B. Conductivitéélectrique membranaire: étude de l'effet de la concentration, de la nature de l'électrolyte et de la structure membranaire. *Eur. Polym. J.* **1999**, *35*, 1187–1195, doi:10.1016/S0014-3057(98)00213-4.
13. Belova, E.I.; Lopatkova, G.Y.; Pismenskaya, N.D.; Nikonenko, V. V.; Larchet, C.; Pourcelly, G. Effect of Anion-exchange Membrane Surface Properties on Mechanisms of Overlimiting Mass Transfer. *J. Phys. Chem. B* **2006**, *110*, 13458–13469, doi:10.1021/jp062433f.
14. Ślęzak, A.; Bryll, A.; Grzegorczyn, S. A Numerical Study of the Hydrodynamic Stable Concentration Boundary Layers in a Membrane System Under Microgravitational Conditions. *J. Biol. Phys.* **2006**, *32*, 553–562, doi:10.1007/s10867-007-9037-0.
15. Porozhnyy, M.; Huguet, P.; Cretin, M.; Safronova, E.; Nikonenko, V. Mathematical modeling of transport properties of proton-exchange membranes containing immobilized nanoparticles. *Int. J. Hydrogen Energy* **2016**, *41*, 15605–15614, doi:10.1016/j.ijhydene.2016.06.057.
16. Lévêque M. A. The laws of heat transmission by convection. *Les Ann. des Mines Mem.* **1928**, *12*, 201–299.
17. Newman J., T.-A.K.E. *Electrochemical systems*; John Wiley & Sons, 2012;
18. N.P. Gnusin, V.I. Zabolotskii, V.V. Nikonenko, M.K.U. Convective-Diffusion Model of Electrodialytic Desalination. Limiting Current and Diffusion Layer. *Sov. Electrochem.* **1986**, *23*, 273–278.
19. La Cerva, M.; Gurreri, L.; Tedesco, M.; Cipollina, A.; Ciofalo, M.; Tamburini, A.; Micale, G. Determination of limiting current density and current efficiency in electrodialysis units. *Desalination* **2018**, *445*, 138–148, doi:10.1016/j.desal.2018.07.028.
20. Nikonenko, V.; Nebavsky, A.; Mareev, S.; Kovalenko, A.; Urtenov, M.; Pourcelly, G. Modelling of Ion Transport in Electromembrane Systems: Impacts of Membrane Bulk and

Surface Heterogeneity. *Appl. Sci.* **2018**, *9*, 25, doi:10.3390/app9010025.

21. Belashova, E.D.; Pismenskaya, N.D.; Nikonenko, V.V.; Sistat, P.; Pourcelly, G. Current-voltage characteristic of anion-exchange membrane in monosodium phosphate solution. Modelling and experiment. *J. Memb. Sci.* **2017**, *542*, 177–185, doi:10.1016/j.memsci.2017.08.002.
22. Helfferich, F.G. *Ion Exchange*; McGraw-Hill, 1962;
23. Urtenov, M.A.K.; Kirillova, E. V.; Seidova, N.M.; Nikonenko, V. V. Decoupling of the Nernst-Planck and Poisson equations. Application to a membrane system at overlimiting currents. *J. Phys. Chem. B* **2007**, doi:10.1021/jp073103d.
24. Kharkats, Y.I.; Sokirko, A.V. Theory of the effect of migration current exaltation taking into account dissociation-recombination reactions. *J. Electroanal. Chem.* **1991**, *303*, 27–44, doi:10.1016/0022-0728(91)85113-4.
25. Titorova, V.D.; Mareev, S.A.; Gorobchenko, A.D.; Gil, V.V.; Nikonenko, V.V.; Sabbatovskii, K.G.; Pismenskaya, N.D. Effect of current-induced coion transfer on the shape of chronopotentiograms of cation-exchange membranes. *J. Memb. Sci.* **2021**, *624*, 119036, doi:10.1016/j.memsci.2020.119036.
26. Lide, D.R. *CRC Handbook of Chemistry and Physics 86TH Edition 2005-2006*; 2005; ISBN 0849304792.
